# Cytokine release syndrome-like serum responses after COVID-19 vaccination are frequent and clinically inapparent under cancer immunotherapy

**DOI:** 10.1038/s43018-022-00398-7

**Published:** 2022-06-17

**Authors:** Thomas Walle, Sunanjay Bajaj, Joscha A. Kraske, Thomas Rösner, Christiane S. Cussigh, Katharina A. Kälber, Lisa Jasmin Müller, Sophia Boyoung Strobel, Jana Burghaus, Stefan M. Kallenberger, Christoph K. Stein-Thöringer, Maximilian Jenzer, Antonia Schubert, Steffen Kahle, Anja Williams, Birgit Hoyler, Lin Zielske, Renate Skatula, Stefanie Sawall, Mathias F. Leber, Russell Z. Kunes, Johannes Krisam, Carlo Fremd, Andreas Schneeweiss, Jürgen Krauss, Leonidas Apostolidis, Anne Katrin Berger, Georg M. Haag, Stefanie Zschäbitz, Niels Halama, Christoph Springfeld, Romy Kirsten, Jessica C. Hassel, Dirk Jäger, NCT ANTICIPATE Investigators, Christiane S. Cussigh, Katharina A. Kälber, Omar Abdelrahim, Elena Busch, Patrick Derigs, Katharina Dischinger, Fouad Mitri, Kerstin Schmidt, Irfan A. Bhatti, Barbara Grün, Nicolas Hohmann, Lena Woydack, Xin-Wen Zhang, Dyke Ferber, Andreas Mock, Tillmann Pompecki, Timo Schank, Carlo Fremd, Georg M. Haag, Niels Halama, Romy Kirsten, Jessica C. Hassel, Dirk Jäger, Guy Ungerechts

**Affiliations:** 1 Clinical Cooperation Unit Virotherapy, German Cancer Research Center (DKFZ), Heidelberg, Germany; 2 Department of Medical Oncology, National Center for Tumor Diseases, Heidelberg University Hospital, Heidelberg, Germany; 3 German Cancer Consortium (DKTK), Heidelberg, Germany; 4 Department of Dermatology, National Center for Tumor Diseases, Heidelberg University Hospital, Heidelberg, Germany; 5 Department of Hematology, University Hospital Heidelberg, Heidelberg, Germany; 6 Division Microbiome and Cancer, German Cancer Research Center (DKFZ), Heidelberg, Germany; 7 BioQuant & Department of Cell and Molecular Biology, Heidelberg University, Heidelberg, Germany; 8 Division Signaling and Functional Genomics, German Cancer Research Center (DKFZ), Heidelberg, Germany; 9 NCT Liquid Biobank, National Center for Tumor Diseases (NCT), Heidelberg, Germany; 10 Department of Statistics, Columbia University, New York, NY USA; 11 Institute of Medical Biometry, Heidelberg University, Heidelberg, Germany; 12 Division of Gynecological Oncology, National Center for Tumor Diseases (NCT), Heidelberg, Germany; 13 Clinical Cooperation Unit Applied Tumor-Immunity, German Cancer Research Center (DKFZ), Heidelberg, Germany; 14 Department of Translational Immunotherapy, German Cancer Research Center (DKFZ), Heidelberg, Germany; 15 Helmholtz Institute for Translational Oncology (HITRON), Mainz, Germany; 16 CanVirex, Heidelberg, Germany; 17 Ottawa Hospital Research Institute, Cancer Therapeutics Program, Ottawa, Ontario Canada; 18 Department of Gastroenterology, Heidelberg University Hospital, Heidelberg, Germany

**Keywords:** SARS virus, Viral infection, Cancer, Cancer immunotherapy

## Abstract

Patients with cancer frequently receive immune-checkpoint inhibitors (ICIs), which may modulate immune responses to COVID-19 vaccines. Recently, cytokine release syndrome (CRS) was observed in a patient with cancer who received BTN162b2 vaccination under ICI treatment. Here, we analyzed adverse events and serum cytokines in patients with 23 different tumors undergoing (*n* = 64) or not undergoing (*n* = 26) COVID-19 vaccination under ICI therapy in a prospectively planned German single-center cohort study (*n* = 220). We did not observe clinically relevant CRS (≥grade 2) after vaccination (95% CI 0–5.6%; Common Terminology of Adverse Events v.5.0) in this small cohort. Within 4 weeks after vaccination, serious adverse events occurred in eight patients (12.5% 95% CI 5.6–23%): six patients were hospitalized due to events common under cancer therapy including immune related adverse events and two patients died due to conditions present before vaccination. Despite absence of CRS symptoms, a set of pairwise-correlated CRS-associated cytokines, including CXCL8 and interleukin-6 was >1.5-fold upregulated in 40% (95% CI 23.9–57.9%) of patients after vaccination. Hence, elevated cytokine levels are common and not sufficient to establish CRS diagnosis.

## Abstract (web summary)

Walle & Bajaj et al. assess the frequency of cytokine release syndrome upon COVID-19 vaccination in patients with cancer receiving immune-checkpoint inhibitors and find that while elevated cytokines levels are common, they do not manifest in detectable CRS.

## Main

Patients with solid tumors have an increased fatality risk after infection with the SARS-CoV-2 coronavirus^1^. Patients with cancer have therefore been prioritized for vaccination against SARS-CoV-2 (COVID-19 vaccination) in many countries^2,3^. Approved vaccines in Europe and the United States use messenger RNA lipid nanoparticles or viral vectors to transiently transfect/transduce a SARS-CoV-2 spike mRNA/transgene, which is translated in the patient’s healthy cells at the site of vaccination, thus strongly inducing cellular and humoral adaptive immunity^4–8^. However, patients with cancer were underrepresented in clinical phase III trials leading to US Food and Drug Administration and European Medicines Agency approval of these vaccines^6,7,9^. Moreover, an increasing number of patients with cancer receive immunomodulatory cancer therapies, mostly ICIs blocking the PD-1–PD-L1 coinhibitory axis for T-cell activation^10^. As ICIs lead to reactivation of tumor antigen-reactive T cells, it is possible that ICIs may also influence activation of SARS-CoV-2 spike protein (S1)-specific T cells. This increased T-cell activation may lead to massive cytokine release and subsequent clinical reactions. The body’s systemic response to the resulting release of multiple inflammatory cytokines from T and myeloid cells is called CRS. CRS manifests itself in fever, hypotension, hypoxia and multiorgan dysfunction at later stages^11^. Most frequently such responses are observed after adoptive T-cell therapies, bispecific antibodies to the CD3 co-receptor or severe infection^12^. CRS is commonly graded according to the Common Terminology of Adverse Events (CTCAE) or the American Society for Transplantation and Cellular Therapy (ASTCT) consensus grading^11,12^; however, fever ≥38 °C alone is sufficient to establish CTCAE grade 1 CRS, which does not account for mild fever as part of many appropriate immune reactions^12^. Hence, an exhaustive differential diagnosis is essential in establishing CRS^12^.

Au et al. recently reported a patient with CRS without evidence for infection after COVID-19 vaccination under ICI therapy^13^. This patient required hospitalization due to fever, thrombocytopenia (grade 3 CTCAE 4.03 and grade 1 ASTCT) as well as increased C-reactive protein (CRP) levels (CRP > 200 mg l^−1^) and CRS-associated cytokine release^13^. This observation prompted questions about the frequency of CRS in patients with cancer under ICI treatment^13^ and whether cytokine profiles could be explored to identify ICI-treated patients with cancer at risk for CRS at early stages.

Here, we assessed adverse events (AEs), clinical laboratory data and serum cytokine responses in patients undergoing combined ICI and COVID-19 vaccination as an exploratory end point of a prospectively registered German single-center cohort study.

## Results

### A pan-tumor cohort study across immune combination therapies

**Fig. 1:**
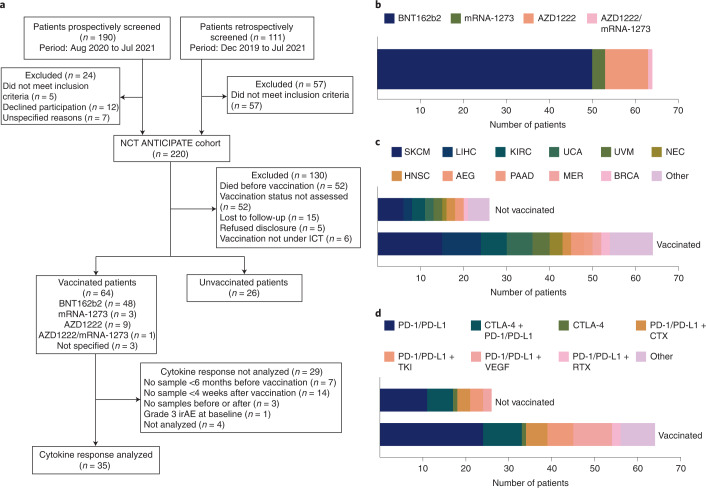
A pan-tumor cohort study across diverse immune combination therapies. **a**, CONSORT flow chart indicating patient screening data and cohorts for subsequent data analysis. **b**, Stacked bar-graph depicting the type of COVID-19 vaccination for vaccinated patients (*n* = 64). **c**, Stacked bar graph indicating tumor types of vaccinated (*n* = 64) and unvaccinated (*n* = 26) patients. AEG, adenocarcinoma of the esophagogastric junction; BRCA, breast cancer; HNSC, head and neck squamous cell carcinoma; KIRC, renal cell carcinoma; LIHC, liver hepatocellular carcinoma; MER, Merkel cell carcinoma; NEC, neuroendocrine carcinoma; PAAD, pancreatic adenocarcinoma; SKCM, skin melanoma; UCA, urothelial carcinoma; UVM, uveal melanoma. **d**, Stacked bar-graph indicating immunotherapies of vaccinated (*n* = 64) and unvaccinated (*n* = 26) patients. CTX, chemotherapy; RTX, radiotherapy; TKI, tyrosine kinase inhibitor. Source data

Between 2 December 2019 and 20 July 2021, we screened 301 patients, of which 190 were screened prospectively and 111 retrospectively as defined in the trial protocol (German Clinical Trials Register, DRKS00022890). We recruited 220 patients with advanced solid tumors undergoing ICI therapy at our center (Fig. 1a). These patients received regular blood sampling before and during therapy and were monitored for AEs. We assessed the COVID-19 vaccination status of the patients within the cohort during treatment follow-up and identified 64 patients who received a COVID-19 vaccine while under ICI therapy and 26 patients who did not (Fig. 1a,b). The remaining patients either died before the vaccine was widely available (*n* = 52), did not have their vaccination status assessed (*n* = 52), were lost to follow-up (*n* = 15), refused to disclose their vaccination status (*n* = 5) or received COVID-19 vaccinations before ICI therapy (*n* = 6) (Fig. 1a).

We focused further analyses on the 90 patients with known vaccination status (Table 1 and Supplementary Table 1). A total of 23 cancer types were represented within this patient group, the most frequent being skin melanoma (*n* = 21), hepatocellular carcinoma (*n* = 11) and renal cell carcinoma (*n* = 9) (Table 1 and Fig. 1c). Our study included fewer female than male patients, a bias possibly resulting from the recruitment of few gynecological malignancies (Table 1). Therapies included a variety of combinatorial immunomodulatory therapies, most frequently anti-PD-1/PD-L1 monotherapy (*n* = 35), combined anti-PD-1 and anti-CTLA-4 therapy (*n* = 15) and a combination of anti-PD-1/PD-L1 with anti-VEGF (*n* = 11) (Table 1 and Fig. 1d). Despite the limited patient sizes of our cohorts, vaccinated and unvaccinated patients showed similar clinical characteristics such as sex, age, tumor type, stage, comorbidities, therapy regimen and line of therapy (Table 1) and were hence deemed suitable for further comparisons.

**Table 1:** Characteristics of analyzed patients at baseline

Characteristic	Vaccinated (*n* = 64)	Unvaccinated (*n* = 26)	Total (*n* = 90)
Patient recruitment characteristics			
Vaccination period (MM/YY–MM/YY)	01/21–07/21	–	01/21–07/21
Follow-up from first vaccine, days (median)	24–259 (169)	–	24–259 (169)
Start of ICI period (MM/YY–MM/YY)	03/20–05/21	07/20–07/21	03/20–07/21
Follow-up from ICI, days (median)	44–574 (254)	30–441 (203.5)	30–574 (229)
Vaccination type, *n* (%)			
BNT162b2	50 (78.1)	-	50 (55.6)
mRNA-1273	3 (4.7)	-	3 (3.3)
AZD1222	10 (15.6)	-	10 (11.1)
AZD1222/mRNA-1273	1 (1.6)	-	1 (1.1)
Sex, *n* (%)			
Male	44 (68.8)	19 (73.1)	63 (70.0)
Female	20 (31.3)	7 (26.9)	27 (30.0)
Age group at treatment, years *n* (%)			
18–60	19 (29.7)	7 (26.9)	26 (28.9)
>60	45 (70.3)	19 (73.1)	64 (71.1)
Insurance type, *n* (%)			
General	55 (85.8)	22 (84.6)	77 (85.6)
Private	9 (14.2)	4 (15.4)	13 (14.4)
ECOG, *n* (%)			
0	11 (17.2)	7 (26.9)	18 (20.0)
1	46 (71.9)	13 (50.0)	59 (65.6)
2	7 (10.9)	4 (15.4)	11 (12.2)
3	0 (0.0)	2 (7.7)	2 (2.2)
4–5	0 (0.0)	0 (0.0)	0 (0.0)
Cancer type, *n* (%)			
Melanoma	15 (23.4)	6 (23.1)	21 (23.3)
Hepatocellular carcinoma	9 (14.1)	2 (7.7)	11 (12.2)
Renal clear cell carcinoma	6 (9.4)	3 (11.5)	9 (10.0)
Urothelial carcinoma	6 (9.4)	2 (7.7)	8 (8.9)
Uveal melanoma	4 (6.3)	2 (7.7)	6 (6.7)
Neuroendocrine carcinoma	3 (4.7)	1 (3.8)	4 (4.4)
H&N squamous carcinoma	2 (3.1)	2 (7.7)	4 (4.4)
Adenocarcinoma of the EG junction	3 (4.7)	2 (7.7)	5 (5.6)
Pancreatic adenocarcinoma	2 (3.1)	–	2 (2.2)
Merkel cell carcinoma	2 (3.1)	–	2 (2.2)
Breast cancer	2 (3.1)	1 (3.8)	3 (3.3)
Other^a^	10 (15.6)	5 (19.2)	15 (16.7)
UICC stage at diagnosis, *n* (%)			
I	8 (12.5)	4 (15.4)	12 (13.3)
II	10 (15.6)	6 (23.1)	16 (17.8)
III	11 (17.2)	8 (30.8)	19 (21.1)
IV	34 (53.1)	7 (26.9)	41 (45.6)
unknown (not IV)	1 (1.6)	1 (3.8)	2 (2.2)
Line of therapy, *n* (%)			
First line	38 (59.3)	15 (57.7)	53 (58.9)
Second line	13 (20.3)	4 (15.4)	17 (18.9)
3–5 line	10 (15.6)	6 (23.1)	16 (17.8)
≥6 line	3 (4.7)	1 (3.8)	4 (4.4)
Other comorbidities, *n* (%)^b^			
Arterial hypertension	29 (45.3)	9 (34.6)	38 (42.2)
Coronary heart disease	8 (12.5)	2 (7.7)	10 (11.1)
Other cardiomyopathy	2 (3.1)	3 (11.5)	5 (5.6)
Cerebrovascular disease/peripheral vascular disease	6 (9.4)	2 (7.7)	8 (8.9)
Chronic pulmonary disease	2 (3.1)	1 (3.8)	3 (3.3)
Diabetes	8 (12.5)	6 (23.1)	14 (15.6)
Other endocrine disorder	12 (18.8)	5 (19.2)	17 (18.9)
Hepatic disease	6 (9.4)	3 (11.5)	9 (10.0)
Renal disease	5 (7.8)	5 (19.2)	10 (11.1)
Rheumatic disease	2 (3.1)	3 (11.5)	5 (5.6)
No comorbidities	4 (6.3)	0 (0)	4 (4.4)
BMI, *n* (%)			
<18.5	1 (1.6)	2 (7.7)	3 (3.3)
≥18.5 to <25.0	29 (45.3)	8 (30.8)	37 (41.1)
≥25.0 to <30.0	13 (20.3)	7 (26.9)	20 (22.2)
≥30.0	8 (12.5)	4 (15.4)	12 (13.3)
NA	13 (20.3)	5 (19.2)	18 (20.0)
Treatment, *n* (%)			
PD-1/PD-L1 mono	24 (37.5)	11 (42.3)	35 (38.9)
CTLA-4 + PD-1/PD-L1	9 (14.1)	6 (23.1)	15 (16.7)
CTLA-4 mono	1 (1.6)	1 (3.8)	2 (2.2)
PD-1/PD-L1 + CTX	5 (7.8)	3 (11.5)	8 (8.9)
PD-1/PD-L1 + TKI	6 (9.4)	3 (11.5)	9 (10.0)
PD-1/PD-L1 + VEGF	9 (14.1)	2 (7.7)	11 (12.2)
PD-1/PD-L1 + RTX	2 (3.1)	–	2 (2.2)
PD-1/PD-L1 + other	8 (12.5)	–	8 (8.9)

^a^Others include ampullary carcinoma, adrenocortical carcinoma, cholangiocarcinoma, carcinoma of unknown primary, dermal squamous cell carcinoma, esophageal squamous cell carcinoma, head and neck mucosal melanoma, kidney renal papillary cell carcinoma, lung adenocarcinoma, neuroendocrine tumor, oral squamous cell carcinoma and renal cell carcinoma.

^b^Number of patients with comorbidity listed. Patients can have multiple comorbidities.

H&N, head and neck; EG, esophagogastric; UICC, Union for International Cancer Control; BMI, body mass index; NA, not available; CTX, chemotherapy; TKI, tyrosine kinase inhibitor; VEGF, vascular endothelial growth factor antibody; RTX, radiotherapy; –, not applicable.

### Clinical CRS is infrequent after COVID-19 vaccination

**Fig. 2:**
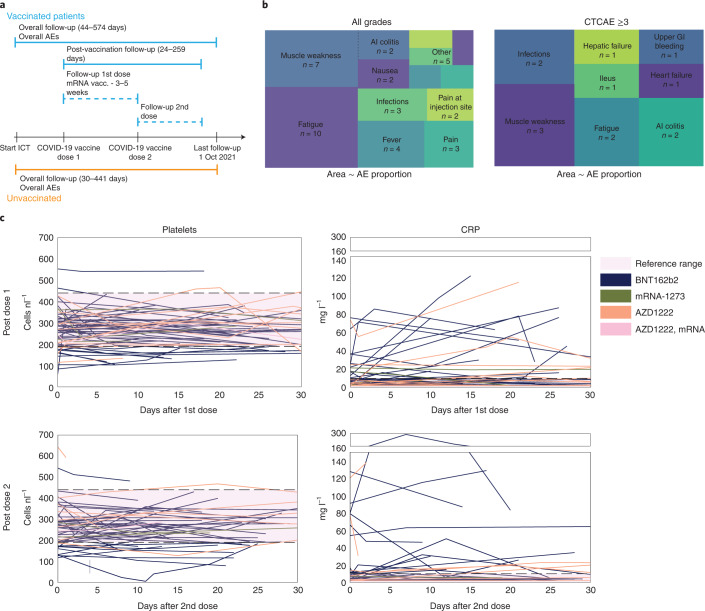
Early adverse events after COVID-19 vaccination under immune-checkpoint therapy. **a**, Schematic timeline depicting the start and duration of follow-up and vaccination time points of vaccinated and unvaccinated patients. **b**, Tree maps visualizing the proportion and the numbers of all and grade ≥3 AEs up to 4 weeks after vaccination in vaccinated patients (*n* = 64). In tree map for all AE: others (*n* = 5) include vomiting (*n* = 1), ileus (*n* = 1), upper GI bleed (*n* = 1), hepatic failure (*n* = 1) and heart failure (*n* = 1); pain (*n* = 3) includes headache (*n* = 2) and arthralgia (*n* = 1); infections (*n* = 3) include *C.* *difficile* infection (*n* = 1), *E.* *coli* sepsis (*n* = 1) and herpes simplex reactivation (*n* = 1). In tree map for grade ≥3 AEs: infections (*n* = 2) include *C.* *difficile* infection (*n* = 1) and *E.* *coli* sepsis (*n* = 1). **c**, Line-plots indicating platelet counts (*n* = 61, left) and CRP (*n* = 55, right) either after the first vaccine dose (top) or second vaccine dose (bottom). Reference ranges are indicated in pink shading. Lines are colored according to the vaccination schema used with the color code indicated below. NA, not available. Source data

To estimate safety of COVID-19 vaccination under ICI therapy, we analyzed early AEs from the first dose until 4 weeks after the second COVID-19 vaccination dose (Fig. 2a, Table 2 and Supplementary Table 2); however, it must be noted that this study was not powered to assess the exact frequency of AEs after vaccination. While fewer early local AEs such as pain at the injection site (*n* = 2, 3.1%) were reported in our cohort, early systemic AEs were comparable to reported AEs in patients with cancer, including patients under ICI therapy^14,15^. The most common systemic AEs included fatigue (*n* = 10, 15.6%), muscle weakness (*n* = 7, 10.9%) and fever (*n* = 4, 6.3%) (Fig. 2b and Table 2). Six patients (9.4%) were hospitalized due to grade ≥3 AEs and two of these patients died (3.1%) (Fig. 2b). These patients are described in detail in the Supplementary Note.

**Table 2:** Early adverse events after COVID-19 vaccination (≤4 weeks)

AE	Any grade	Grade ≥3
Any AE, *n* (%)	39 (60.9)	8 (12.5)
No AE, *n* (%)	25 (39.1)	56 (87.5)
AEt, *n* (%)		
Fatigue	10 (15.6)	2 (3.1)
Pain at injection site	2 (3.1)	0 (0.0)
Muscle weakness	7 (10.9)	3 (4.7)
Fever	4 (6.3)	0 (0.0)
Infections^a^	3 (4.7)	2 (3.1)
Nausea	2 (3.1)	0 (0.0)
Pain^b^	3 (4.7)	0 (0.0)
Vomiting	1 (1.6)	0 (0.0)
Upper GI bleeding	1 (1.6)	1 (1.6)
Ileus	1 (1.6)	1 (1.6)
AI colitis	2 (3.1)	2 (3.1)
Hepatic failure	1 (1.6)	1 (1.6)
Heart failure	1 (1.6)	1 (1.6)
CRS grade ≥2	0 (0)	0 (0)
Laboratory abnormalities, *n* (%)		
Anemia	7 (10.9)	1 (1.6)
Thrombocytopenia	8 (12.5)	1 (1.6)
Bilirubin increase	8 (12.5)	0 (0)
Creatinine increase	6 (9.4)	1 (1.6)
Alanine transaminase increase	1 (1.6)	1 (1.6)
Aspartate transaminase increase	1 (1.6)	1 (1.6)
Death, *n* (%)	2 (3.1)	

^a^Infections, *C.* *difficile* diarrhea (*n* = 1), herpes simplex reactivation (*n* = 1) and *E.* *coli* sepsis (*n* = 1)

^b^Pain, headache (*n* = 2) and arthralgia (*n* = 1). GI, gastrointestinal; AI, autoimmune.

One patient who received the BNT162b2 vaccine was admitted after the first COVID-19 vaccination for autoimmune colitis, which resolved under intravenous (i.v.) glucocorticoids with subsequent oral tapering (Supplementary Note). The patient was also admitted with grade 4 anemia due to esophageal varices bleeding after the second COVID-19 vaccination and recovered quickly under high-dose proton pump-inhibitor therapy. During this stay the patient also experienced a brief febrile (38–39 °C) episode of 2 d for which no infectious focus could be established. Although the patient did not show hypotension or hypoxia and recovered after 2 d on ampicillin/sulbactam, we cannot fully exclude the possibility that vaccination contributed to this febrile episode. The second patient experienced grade 3 increase of transaminases under pembrolizumab + axitinib and the mRNA-1273 vaccine, which normalized within 3 weeks after initial i.v. methylprednisolone and subsequent oral glucocorticoid tapering (Supplementary Note). The third patient already exhibited grade 2 diarrhea before BNT162b2 vaccination which worsened to grade 3 2 weeks after vaccination (Supplementary Note). Multiplex-PCR analyses of stool showed *Clostridium* *difficile* and symptoms improved after therapy with i.v. fluids and antibiotics. The fourth patient received the BNT162b2 vaccine and was admitted due to grade 3 diarrhea for which no infectious cause could be determined and was therefore deemed to be ICI-related (Supplementary Note). Symptoms resolved under i.v. fluids and corticosteroids with oral tapering. One patient died from hepatic failure after computed tomography (CT)-confirmed fulminant hepatic disease progression causing cholestasis (Supplementary Note). Finally, one patient with a history of combined severe aortic stenosis (0.8 cm^2^ aortic valve area) and aortic insufficiency, who had paused all cardiac medication against the treating physician’s advice, died at home after cardiac decompensation with pleural effusion and lower limb edema after AZD1222 administration (Supplementary Note).

We observed fever in several patients after vaccination (6.25%), which has also been observed in phase 3 trials leading to vaccine approval in patients without cancer (Fig. 2b and Table 2)^7,9,16^. We cannot fully exclude the possibly of grade I CRS in these patients, which can manifest as fever alone according to the ASTCT^11^ or CTCAE v.5.0 criteria predefined in the study protocol; however, we observed no hypotension or hypoxia in any febrile patient and hence no CRS ≥ grade II (Fig. 2b and Supplementary Tables 2 and 3). Despite this observation, it is theoretically possible that cytokine release may have contributed to some AEs. In the above-mentioned case report, CRS was associated with thrombocytopenia and CRP increase^13^. In our cohort, only one patient experienced grade ≥3 thrombocytopenia with a platelet count of 5 nl^−1^ 4 d after the second BNT162b2 dose (Fig. 2c). This patient had received gemcitabine and carboplatin 3 d before the event while still under prednisolone (50 mg d^−1^) due to a grade 3 autoimmune hemolytic anemia, which started after a blood transfusion 2 months earlier. The patient was asymptomatic, afebrile and was not hospitalized. Moreover, platelet counts spontaneously normalized within 2 weeks thus making CRS unlikely (Fig. 2c). We frequently observed mild (>30 mg l^−1^ and >1.5-fold) CRP increase after vaccination (*n* = 22, 40% after first dose; *n* = 17, 35% after second dose; Fig. 2c). One patient showed a severe CRP increase (80−289 mg l^−1^) peaking 7 d after the second BNT162b2 dose (Fig. 2c). This patient was also asymptomatic, including absence of fever/hypotension or hypoxia, thus making CRS unlikely. Blood and urine cultures remained negative and CRP spontaneously dropped below 100 mg l^−1^ within 2 weeks. Hence, we did not observe any clinically apparent CRS after COVID-19 vaccination in our cohort, suggesting that CRS may be rare in patients under ICI therapy with concurrent COVID-19 vaccination.

### CRS-like cytokine release after COVID-19 vaccination

**Fig. 3:**
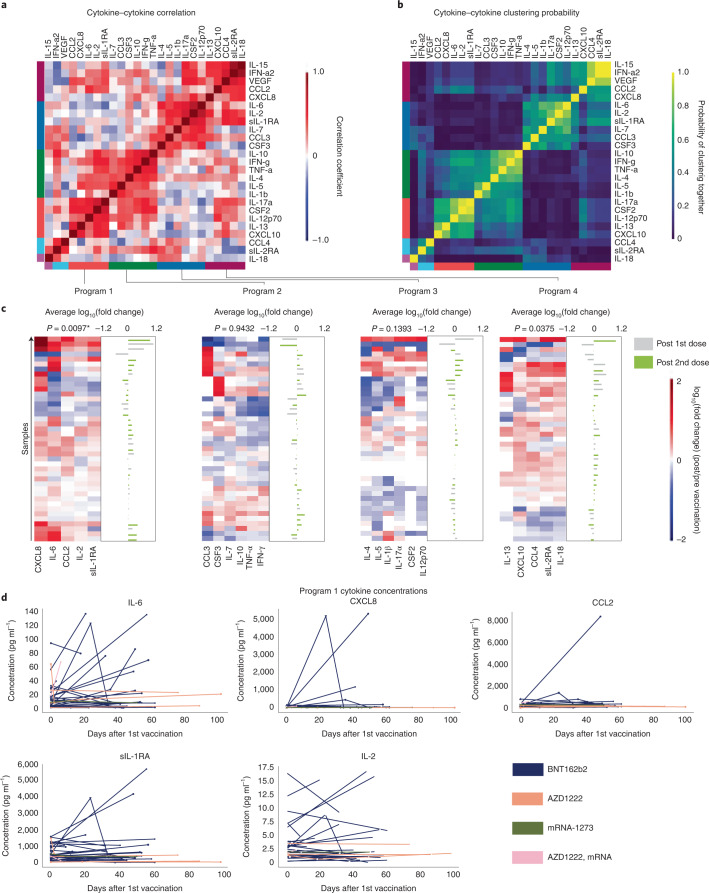
A correlated program of CRS-related cytokines is frequently upregulated after COVID-19 vaccination under immune-checkpoint therapy. **a**, Heat map indicating Pearson correlation indices of log_10_(fold change) cytokine concentrations after COVID-19 vaccination from *n* = 35 patients. Colored bars on the sides indicate clusters obtained from hierarchical clustering. **b**, Heat map indicating probability of each pair of cytokines clustering together as calculated by bootstrapping (*n* = 10,000 samplings) from *n* = 35 patients. Colored bars on the sides indicate clusters obtained from hierarchical clustering. **c**, Heat maps indicating log_10_(fold change) of cytokine concentrations after vaccination. Bar graph on the side indicates average log_10_(fold change) of cytokines in each row with concentrations after the first or second vaccination dose labeled according to the color code on the right from *n* = 35 patients. *P* values (two-sided) were calculated using a Wilcoxon signed-rank test (test statistics from left to right are 231, 425, 301 and 270; Cohen’s D calculated as mean fold change/s.d. fold change from left to right is 0.93, 3.22, 1.32 and 1.21). *P* values significant (α = 0.05) after correcting for multiple comparisons with the Benjamini–Hochberg method are indicated with an asterisk. **d**, Line-plots indicating cytokine concentrations of cytokine program 1 cytokines after vaccination from *n* = 35 patients and vaccine type indicated in the color code at the bottom right. Source data

**Extended Data Fig. 1:**
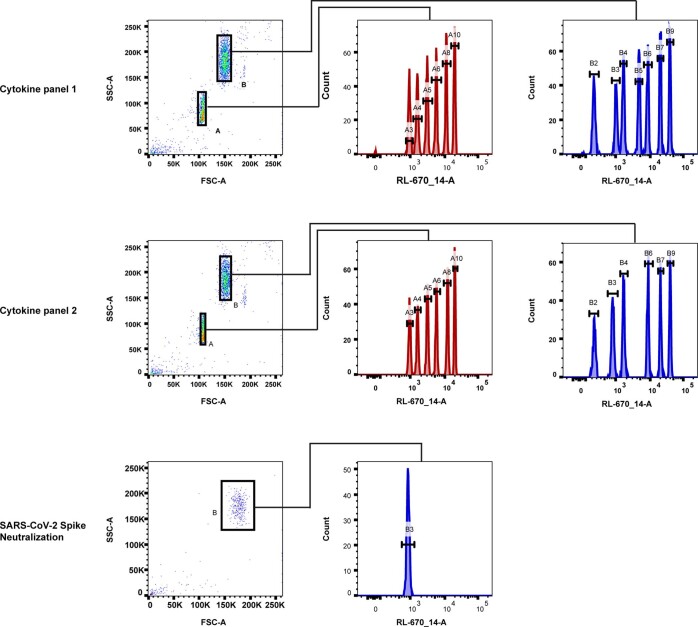
Gating strategy for flow-cytometry based multiplex cytokine array, related to Fig. 3. Schema outlining the gating strategy used in the multiplex cytokine arrays and neutralizing antibody assay to define bead populations based on sideward-scatter (SSC), forward-scatter (FSC) and fluorescence signal in the red laser 670/14 channel. First row indicating gating schemas for cytokine panel 1 (741091, Biolegend, CA, USA), second row indicating gating for cytokine panel 2 (741142, Biolegend, CA, USA) and third row indicating gating for SARS-CoV-2 Neutralizing Antibody Assay (741127, Biolegend, CA, USA).

To evaluate cytokine responses indicative of CRS, we analyzed serum levels of CRS-associated cytokines in 35 patients undergoing concurrent ICI therapy and COVID-19 vaccination with a baseline sample ≤6 months before vaccination and a sample ≤6 weeks after vaccination (Fig. 3a,b and Extended Data Fig. 1). We excluded one patient who had an immune-related AE (arthritis grade 3) at baseline before vaccination.

**Extended Data Fig. 2:**
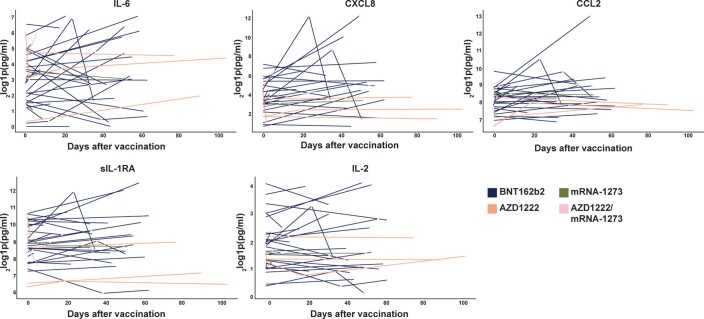
Cytokine concentrations in COVID-19 vaccinated patients, related to Fig. 3. Line-plots indicating log1p transformed individual cytokine concentrations of cytokine program 1 cytokines after vaccination in n = 35 patients. log1p(x) = _10_log(x + 1) with x: cytokine concentration in pg/ml. Source data

**Extended Data Fig. 3:**
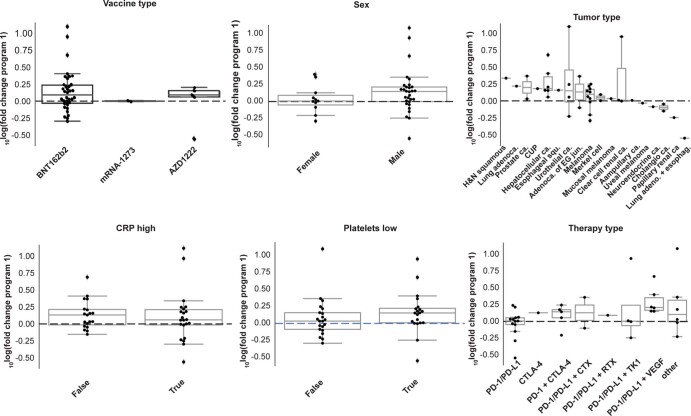
Cytokine program 1 upregulation stratified by patient characteristics, related to Fig. 3. Box-plots indicating median _10_log(fold change) of cytokine program 1 after COVID-19 vaccination (horizontal lines) with inter-quartile range (boxes) and range (whiskers, range calculation not including outliers as implemented in seaborn.boxplot function) stratified according to vaccine type (left upper panel), sex (middle upper panel), tumor type (right upper panel), above or below median of maximum CRP after vaccination(after 1^st^ vaccination dose for cytokine samples after 1^st^ vaccination or after 2^nd^ vaccination dose for cytokine samples after 2^nd^ vaccination), above or below median of minimum platelet counts after vaccination (after 1^st^ vaccination dose for cytokine samples after 1^st^ vaccination or after 2^nd^ vaccination dose for cytokine samples after 2^nd^ vaccination) or by therapy type (n = 41 patient samples). Source data

**Extended Data Fig. 4:**
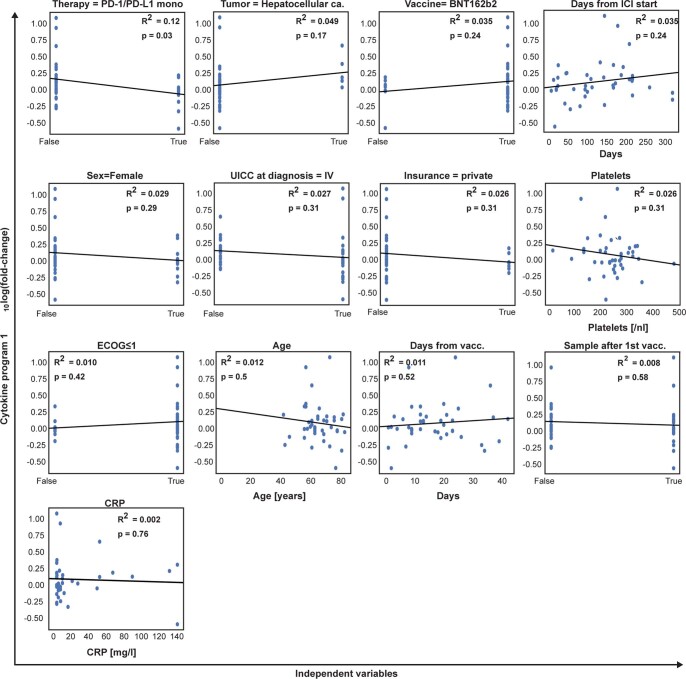
Univariate linear regression for cytokine program 1, related to Fig. 3. Scatter plots indicating average _10_log(fold change) of cytokine program 1 after COVID-19 vaccination (dependent variable) in relation to different clinical variables (independent variables). Boolean variables True and False on the x axis refer to the variable indicated in the header. Lines indicate the regressed function with p values (two-sided) not corrected for multiple comparisons and R^2^ values indicated in each panel (n = 41 patient samples). Source data

To analyze cytokines induced by vaccination under ICI therapy, we performed pairwise correlation of all measured cytokines, which yielded four clusters of pairwise-correlated cytokine programs (colored sidebars; Fig. 3a). To assess the stability of this clustering we bootstrapped the probability of each pair of cytokines falling into the same cluster (Methods). Bootstrapping confirmed the stability of cytokine program 1, whereas the other clusters were more heterogeneous (Fig. 3b). Assessment of the log(fold change) of cytokine concentrations indicated that cytokine programs 1 and 4 were upregulated after vaccination in most patients although only program 1 was statistically significant (*P* = 0.0097, *q* = 0.0389 (false discovery rate)) likely due to the limited sample size and power (Fig. 3c). Program 4 (*P* = 0.0375, *q* = 0.075) included mediators previously described in CRS after the BNT162b2 vaccine in an anti-PD-1 treated patient with cancer such as interleukin (IL)-18 and sIL-2RA^13^. Program 1 included hallmark CRS cytokines and CRS mediators indicative of T-cell (IL-2) and myeloid cell activation (IL-6 and CXCL8 (IL-8, CCL2 and sIL-1R); Fig. 3d and Extended Data Fig. 2). IL-6 has been linked to CRS severity in multiple studies and surpassed 50 pg ml^−1^ (research grade measurement) in eight patients (22.2%), levels frequently observed in patients with severe COVID-19 or CAR-T-cell-induced CRS^17–19^ (Fig. 3d). Stratification by patient characteristics revealed that cytokine program 1 was predominantly upregulated after BNT162b2 vaccination and after combination immunotherapy, especially in patients with hepatocellular carcinoma treated with atezolizumab/bevacizumab (Extended Data Fig. 3); however, these changes were statistically non-significant. Most patients received the BNT162b2 vaccine and univariate linear regression suggested that these features only weakly predicted the increase in cytokine program 1 observed in our patient cohort (Extended Data Fig. 4). To explore clinical correlates of these changes we analyzed the AEs in the ten patients with the highest induction of cytokine program 1 (fold change ≥1.7; Table 3). These patients showed some grade I AEs such as fatigue and arthralgia but no higher-grade AEs or fever, the CRS-defining symptom according to CTCAE or ASTCT (Table 3). All these patients were alive at the end of follow-up (Table 3). Overall, our results suggest induction of CRS-related cytokines as a frequent event after COVID-19 vaccination. This induction, however, does not seem to routinely result in CRS symptoms in the ICI-treated patients with cancer in our study.

**Table 3:** Adverse events in patients with highest induction of cytokine program 1

Patient ID	Fold change cytokine	First vaccination	Second vaccination		Cytokine date	AE	AE date	CTCAE grade	Survival	Follow-up from first vaccination (days)
BWW38Q	12.6	4 Jun 21		2 Jun 21	28 Jun 21	None	NA	NA	Alive	119
0ZTE2L	9.0	15 Apr 21		27 May 21	04 Jun 21	None	NA	NA	Alive	169
2JJ523	4.8	5 May 21		12 Jun 21	10 Jun 21	Arthralgia	13.06.2021	1	Alive	111
1K9IAN	2.5	30 Apr 21		4 Jun 21	24 Jun 21	None	NA	NA	Alive	154
EMHL0E	2.3	27 May 21		8 Jul 21	20 Jul 21	None	NA	NA	Alive	127
SO7XOB	2.3	14 Apr 21		26 May 21	4 Jun 21	Thrombocytopenia	17.06.2021	1	Alive	170
X9H3PX	2.2	28 Apr 21		31 May 21	25 Jun 21	Bilirubin increase	04.06.2021	1	Alive	156
LIK08H	1.8	5 Mar 21		26 Mar 21	7 Apr 21	Fatigue	26.03.2021	1	Alive	210
LIK08H	1.8	5 Mar 21		26 Mar 21	7 Apr 21	General muscle weakness	26.03.2021	1	Alive	210
4WTCUA	1.8	21 Apr 21		31 May 21	12 May 21	Fatigue	31.05.2021	1	Alive	163
X65WDO	1.7	12 Apr 21		13 May 21	25 Jun 21	None	NA	NA	Alive	172

Table shows AEs and CTCAE v.5.0 grading, mean fold change increase in cytokine program 1 and survival data of the ten patients with the highest mean fold change of serum cytokine levels of cytokines IL-6, CXCL8, IL-2, CCL2 and sIL-1R within 4 weeks after vaccination.

### Adverse events after COVID-19 vaccination under ICI

**Fig. 4:**
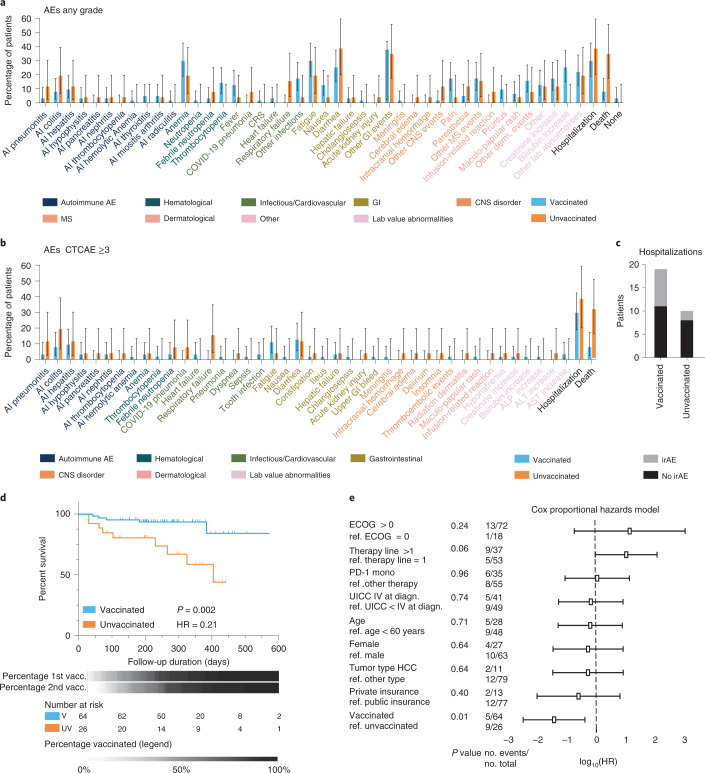
Comparable adverse events and prolonged overall survival in COVID-19-vaccinated immune-checkpoint therapy-treated patients with cancer. **a,b**, Grouped bar plots indicating the frequency and error bars the 95% CI of overall AE (**a**) or grade ≥3 AEs (**b**) under ICI therapy in vaccinated (*n* = 64) and unvaccinated (*n* = 26) patients. This is proportional data and the CI is asymmetric and the center is the bar. **c**, Total number of hospitalizations due to irAEs and other AEs in vaccinated and unvaccinated patients (as listed in Supplementary Table 4) in vaccinated (*n* = 19) and unvaccinated (*n* = 10) patients. **d**, Kaplan–Meier curve indicating overall survival probability of vaccinated (*n* = 64) and unvaccinated (*n* = 26) patients under ICI therapy. *P* values (two-sided, test statistic 9.345) and hazard ratios (95% CI = 0.07–0.69) were calculated using a log-rank test. Grey gradient bars indicate the proportion of patients who received the first or second vaccination dose over time with the timeline indicated above and color legend indicated below. **e**, Forest plot indicating the results of the Cox proportional hazards model of *n* = 90 patients with squares indicating the log_10_(HR) and whiskers indicating the 95% CI. *P* values (two-sided) were not corrected for multiple comparisons and number of events are indicated on the left. HR, hazard ratio; CNS, central nervous system; MS, musculoskeletal. Source data

**Extended Data Fig. 5:**
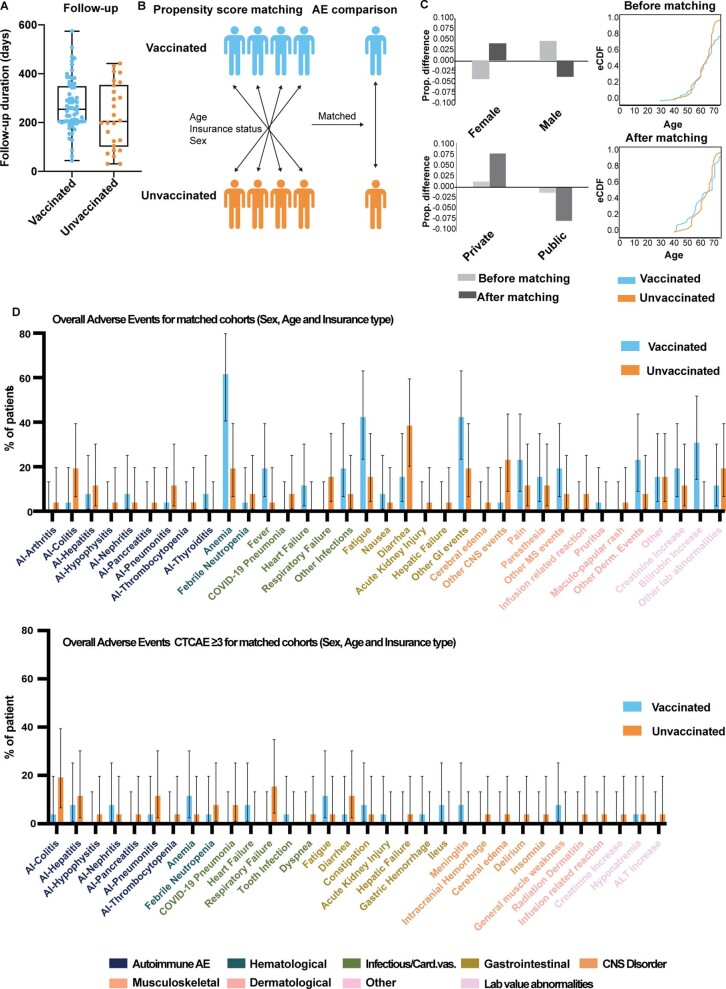
Adverse events in sex, age, insurance type matched COVID-19-vaccinated and unvaccinated immune checkpoint therapy treated patients with cancer, related to Fig. 4. (**a**) Box and whiskers plot depicting the total follow-up duration (days) for vaccinated (n = 64) and unvaccinated (n = 26) patients. The whiskers indicate range, the box the upper and lower quartile and the horizontal bar the median. (**b**) Schema indicating the propensity score based matching procedure. (**c**) Bar graphs indicating the proportional difference of sex and insurance status before and after propensity score matching (upper panel, n = 26 unvaccinated and n = 26 matched vaccinated patients). Xy plot indicating the empirical cumulative distribution function (eCDF) of vaccinated (n = 26) and unvaccinated (n = 26) patients before and after matching. (**d**) Bar graphs indicating frequencies and error bars the 95% confidence interval of all (upper panel) or grade ≥3 (lower panel) adverse events in matched vaccinated (n = 26) and unvaccinated (n = 26) patients. This is proportional data therefore the confidence interval is asymmetric and the center is the bar. Source data

**Extended Data Fig. 6:**
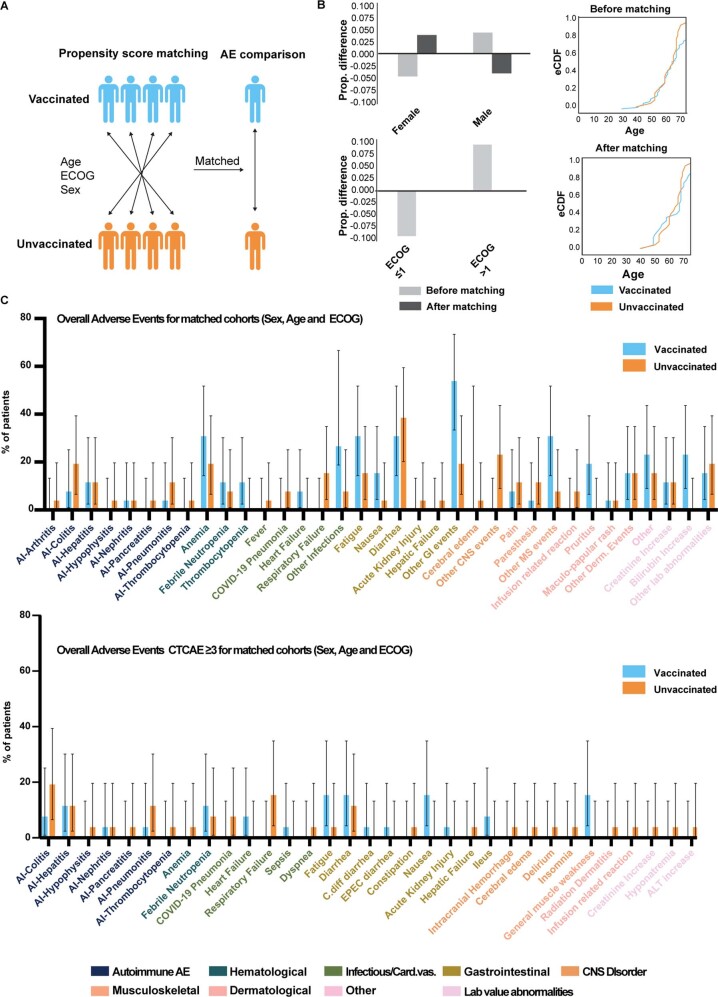
Adverse events in sex, age, ECOG matched COVID-19-vaccinated and unvaccinated immune checkpoint therapy treated cancer patients, related to Fig. 4. (**a**) Schema indicating the propensity score based matching procedure. (**b**) Bar graphs indicating the proportional difference of sex and ECOG before and after propensity score matching (upper panel, n = 26 unvaccinated and n = 26 matched vaccinated patients). Xy plot indicating the empirical cumulative distribution function (eCDF) of vaccinated (n = 26) and unvaccinated (n = 26) patients before and after matching. (**c**) Bar graphs indicating frequencies and error bars the 95% confidence interval of all (upper panel) or grade ≥3 (lower panel) adverse events in matched vaccinated (n = 26) and unvaccinated (n = 26) patients. This is proportional data therefore the confidence interval is asymmetric and the center is the bar. Source data

**Extended Data Fig. 7:**
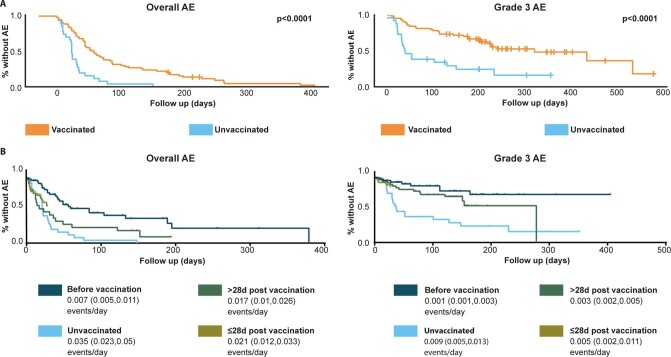
Time to adverse event analysis, related to Fig. 4. (**a**) Kaplan-Meier curves indicating the time without any (left panel) or ≥ CTCAE °3 adverse event (right panel) for vaccinated (n = 64) and unvaccinated patients (n = 26) over the entire course of immune checkpoint therapy with the legend indicated below. P values (two-sided) were calculated using logrank tests (any AE: test statistic= 19.4, grade ≥3 AE: test statistic =18.7). Exact p values: 1.0475761018388364e-05 (left panel), 1.5047888131226484e-05 (right panel). (**b**) Kaplan-Meier curves indicating the time without any (left panel) or ≥ CTCAE °3 adverse event (right panel) for unvaccinated patients (n = 26) over the entire course of immune checkpoint therapy and for vaccinated patients before (n = 48) vaccination, up to 28 days (n = 21) after vaccination and longer than 28 days (n = 37) after vaccination. The color code, average events per day and their 95% confidence intervals (in parenthesis) are indicated in the legend below. Source data

To assess whether vaccination increased the frequency of ICI-related AEs at later time points we compared AEs and hospitalization frequencies in vaccinated (*n* = 64) and unvaccinated patients (*n* = 26) over the entire follow-up period (Extended Data Fig. 5a and Supplementary Tables 3 and 4). We did not detect any significant differences in any grade or grade ≥3 AEs between vaccinated and unvaccinated patients under ICI therapy (Fig. 4a,b). One patient experienced grade 2 CRS before COVID-19 vaccination but no patient showed CRS after vaccination. Immune-related AEs were more frequent in unvaccinated patients, whereas vaccinated patients had a higher frequency of fatigue, nausea and lower grade thrombocytopenia, bilirubin increases and infections (Fig. 4a,b and Table 4); however, more vaccinated patients were hospitalized due to immune-related AEs (irAEs) (8 of 19) compared to unvaccinated patients (2 of 10; Fig. 4c). To confirm the accuracy of these comparisons, we calculated propensity scores based on age-, sex- and insurance status-matched (Extended Data Fig. 5b–d) as well as on age-, sex- and Eastern Cooperative Oncology Group (ECOG)-matched vaccinated and unvaccinated patients (Extended Data Fig. 6a–c). Again, overall and grade ≥3 AEs were comparable between the matched cohorts (Extended Data Figs. 5b–d and 6a–c) suggesting that it is unlikely that COVID-19 vaccination profoundly increased the incidence of severe AEs in ICI-treated patients with cancer. To further characterize the propensity for AEs in our patient cohort, we analyzed the time to first overall or time to first ≥grade 3 AE in unvaccinated and vaccinated individuals (Extended Data Fig. 7). When considering the entire period under immune-checkpoint therapy the propensity for AEs (both overall and ≥grade 3 AE) was higher in unvaccinated patients compared to vaccinated patients (Extended Data Fig. 7a). To investigate the temporal sequence of AEs and vaccination, we analyzed time to AE in the time periods before, immediately after (≤28 d) and at later time periods (>28 d) after vaccination (Extended Data Fig. 7b). We observed higher propensity for AEs in the immediate post-vaccination and late post-vaccination periods compared to the before-vaccination period (Extended Data Fig. 7b); however, the AE propensity in the post-vaccination periods was still lower than in unvaccinated patients. Overall, this analysis suggests that the vaccinated patient cohort had a lower AE propensity than the unvaccinated cohort across the entire ICI therapy period. This observation is unlikely to be caused by the vaccination itself but may be due to other differences in the vaccinated and unvaccinated cohort.

**Table 4:** Immune-related adverse events under immunotherapy in vaccinated and non-vaccinated patients

Characteristic, *n* (%, 95% CI)	Vaccinated (*n* = 64)	Unvaccinated (*n* = 26)
Any AE (irAE + non-irAE)	**62 (96.9, 89.2**–**99.6)**	25 (96.2, 80.4–1.0)
Grade ≥3 (irAE + non-irAE)	31 (48.4, 35.8–61.3)	**18 (69.2, 48.2**–**85.7)**
AI pneumonitis	2 (3.1, 0.4–10.8)	**3 (11.5, 2.4**–**30.2)**
AI colitis	5 (7.8, 2.6–17.3)	**5 (19.2, 6.6**–**39.4)**
AI hepatitis	6 (9.4, 3.5–19.3)	**3 (11.5, 2.4**–**30.2)**
AI hypophysitis	**3 (4.7, 1.0**–**13.1)**	1 (3.9, 0.1–19.6)
AI pancreatitis	0 (0.0, 0.0–5.6)	**1 (3.9, 0.1**–**19.6)**
AI nephritis	2 (3.1, 0.4–10.8)	**1 (3.9, 0.1**–**19.6)**
AI thrombocytopenia	0 (0.0, 0.0–5.6)	**1 (3.9, 0.1**–**19.6)**
AI hemolytic anemia	**1 (1.6, 0.0**–**8.4)**	0 (0.0, 0–13.2)
AI thyroiditis	**3 (4.7, 1.0**–**13.1)**	0 (0.0, 0–13.2)
AI myositis/arthritis	**3 (4.7, 1.0**–**13.1)**	1 (3.9, 0.1–19.6)
AI radiculitis	**1 (1.6, 0.0**–**8.4)**	0 (0.0, 0–13.2)

Table indicating absolute numbers, frequencies and 95% CI of irAEs. The study has not been powered to evaluate the exact frequency of rare irAEs under ICI therapy and COVID-19 vaccination. The irAEs that are numerically more frequent in either the vaccinated or non-vaccinated group are marked in bold font.

**Extended Data Fig. 8:**
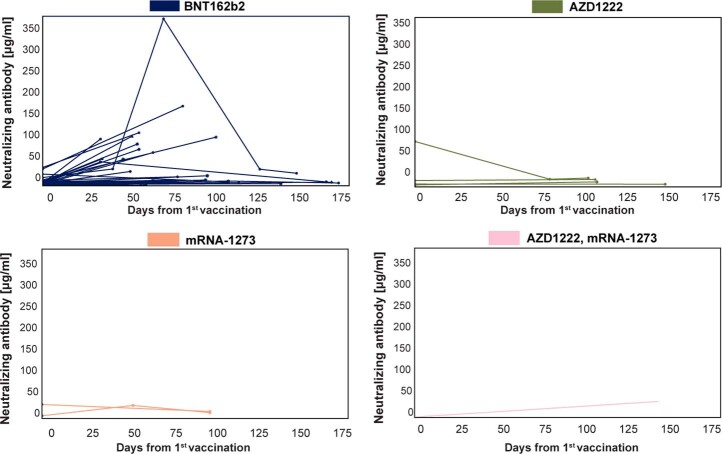
S1 neutralizing antibody concentrations after COVID-19 vaccination in ICI-treated patients, related to Fig. 4. Line-plots indicating neutralizing antibody concentrations of n = 40 individual patients after vaccination as assessed by an ACE2 S1 competitive immunoassay (research grade). Color code indicating vaccine type in the respective patients. Source data

Starting from 15 October 2020, all patients were screened for COVID-19 at every therapy session (every 1–4 weeks) using a rapid antigen test fulfilling the quality criteria of the German Federal Institute for Vaccines and Biomedicines. We detected two COVID-19 patients in the unvaccinated cohort (7.7%, 95% CI 1.6–22.5%) who had to be hospitalized for severe pneumonia (Supplementary Table 4). One patient recovered and was able to resume therapy 6 weeks later but died 2 months after therapy resumption due to disease progression (Supplementary Table 5). The other patient died from COVID-19 pneumonia on the intensive care unit (Supplementary Table 5). We detected no COVID-19 cases in the vaccinated patient cohort, despite regular screening (95% CI 0–5.6%). Most patients were vaccinated with BNT162b2 and patient serum post-BNT162b2 vaccination neutralized SARS-CoV-2 S1 protein binding to recombinant human ACE2 in a competitive immunoassay (Extended Data Fig. 8). Hence, our results corroborate the increasing evidence that the here investigated COVID-19 vaccines have clinically meaningful activity in ICI-treated patients with cancer^8,15^.

**Extended Data Fig. 9:**
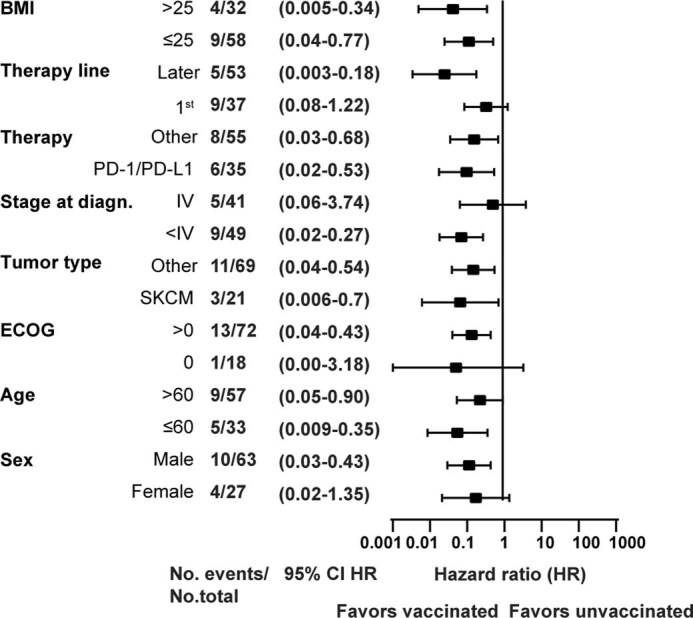
Vaccination is associated with prolonged overall survival probability across patient subgroups, related to Fig. 4. Forest-plot indicating hazard ratios (HR, squares) and 95% confidence intervals (CI, whiskers) of vaccinated (n = 26) and unvaccinated (n = 64) patients stratified into the subgroups indicated with the exact number (no.) of events and patients at risk for each subgroup as well as the 95% CIs indicated on the left. Hazard ratios and confidence intervals were calculated using a Log-rank test or if no events were observed in one group with the Mantel-Hanszel method. sex - female: test statistic = 3.2, sex - male: test statistic = 11.3, age ≤60: test statistic = 9.4, age >60: test statistic = 4.4, ECOG 0: test statistic = 2.0, ECOG > 0: test statistic = 10.6, tumor type = melanoma: test statistic = 5.1, tumor type = other: test statistic = 11.1, stage <4: test statistic = 14.9, stage 4: test statistic = 0.7, therapy = PD-1/PD-L1 mono: test statistic = 7.1, therapy = other: test statistic = 7.0, therapy line = 1st: test statistic = 3.1, therapy line = other: test statistic = 13.5, BMI > 25: test statistic = 8.8, BMI ≤ 25: test statistic = 8.2. Diagn.: diagnosis. Source data

**Extended Data Fig. 10:**
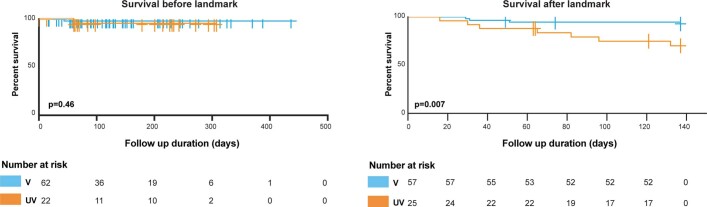
Landmark survival analysis in COVID-19 vaccinated and unvaccinated patients, related to Fig. 4. Kaplan-Meier curves indicating overall survival of COVID-19 vaccinated and unvaccinated immune checkpoint inhibitor treated cancer patients. Survival was analyzed before (left panel) or after (right panel) said landmark. Only patients who gave informed consent until the landmark were included in this analysis. The landmark was defined as 17^th^ May 2020, the date of general eligibility for COVID-19 vaccination for the entire adult population in the state of Baden-Württemberg, Germany. P values (two-sided) were calculated using logrank tests (before landmark: test statistic = 0.5 n = 22 unvaccinated, n = 62 vaccinated, after landmark: test statistic = 7.1 n = 25 unvaccinated, n = 57 vaccinated). Source data

To explore whether vaccination status was associated with oncological outcomes, we compared overall survival of vaccinated and unvaccinated patients (Fig. 4d). Vaccinated patients showed prolonged survival compared to unvaccinated patients (HR 0.21, *P* = 0.002) (Fig. 4d). This effect could not be explained by the single COVID-19 related death we observed among unvaccinated patients (Supplementary Table 5) and was stable across patient subgroups (Extended Data Fig. 9). Moreover, we confirmed this result in a Cox proportional hazards model, where vaccination status was the strongest predictor of prolonged overall survival (coefficient −1.72 95% CI −2.97 to −0.46; *P* = 0.01) (Fig. 4e). As patients were recruited at the start of ICI therapy and not randomized to vaccination, our survival analyses may be subject to guarantee-time bias^20^. We therefore performed a landmark analysis with the landmark set to 17 May 2020, the date of general eligibility for vaccination in the state of Baden-Württemberg (Germany). Before this landmark, high-risk populations, including patients with cancer, had prioritized access to COVID-19 vaccines and consequently 91.9% of all patients vaccinated in this study received their vaccination up to this date. We found that survival of vaccinated patients differed from non-vaccinated patients after but not before the vaccination landmark (Extended Data Fig. 10), further supporting an association of vaccination status with prolonged overall survival in our study. Hence, these data suggest an unexpected association of COVID-19 vaccination and prolonged overall survival in our patient cohort.

## Discussion

In this prospectively planned cohort study, we describe a set of CRS-related cytokines commonly upregulated after COVID-19 vaccination in ICI-treated patients with cancer. None of these patients displayed symptoms of clinically relevant CRS, suggesting that CRS-associated cytokines are frequently induced but rarely symptomatic after COVID-19 vaccination under ICI therapy. Moreover, comparison to unvaccinated patients suggested that COVID-19 vaccination does not profoundly increase the rate of immune-related or grade ≥3 AEs and may decrease the rate of COVID-19 infection in ICI-treated patients.

A recent case report of CRS after vaccination in a patient with colorectal cancer (CRC) treated with a PD-1 ICI highlighted the insufficient evidence regarding vaccine-related AEs in ICI-treated patients with cancer^13^. Au and colleagues presented a patient with fever, thrombocytopenia, CRP increase and elevation of several cytokines including interferon-γ, sIL-2R, IL-18, IL-16 and IL-10 after vaccination and ICI therapy compared to cytokine levels before initiation of ICI therapy and vaccination^13^. In our study we did not observe any CRS ≥ grade II (95% CI 0–5.6%) after COVID-19 vaccination after both short-term (first dose until 4 weeks after second dose) and long-term (median follow-up of 24 weeks after first dose) follow-up according to the ASTCT or CTCAE v.5.0 criteria. Overall, AEs under immunotherapy were generally comparable in vaccinated (48.4% grade ≥3) and unvaccinated patients (69.2% grade ≥3). Moreover, these AE frequencies were within the range of AE frequencies reported in phase 3 clinical trials of the investigated immunotherapies (Supplementary Table 6). Thus, CRS is likely an infrequent event under combined ICI therapy and COVID-19 vaccination.

Despite the absence of clinically relevant CRS, we observed induction of a set of CRS-related cytokines after COVID-19 vaccination under ICI therapy. This included the CRS hallmark cytokine IL-6 and other CRS-related cytokines (CXCL8, IL-2, CCL2 and sIL1-RA). Induction of IL-6 has been reported after mRNA-based lipoplex tumor vaccination which was associated with generally mild and self-limiting associated symptoms^21^. Moreover, we found higher IL-2 levels after vaccination, which may be explained by T-cell activation and preferable type 1 helper T-cell polarization as shown in healthy adults vaccinated with BNT162b2 (ref. ^22^). Our patients also showed coordinated release of CCL2 and CXCL8 levels after COVID-19 vaccination, which can be explained by the activation of myeloid cells by the mRNA-loaded lipid nanoparticles of the BNT162b2 vaccine^23^. While CCL2 and IL-2 were also reported to be induced in the above-mentioned case report of CRS in a patient with mismatch repair-deficient CRC, the CRS hallmark cytokines IL-6 and CXCL8 levels remained largely constant in this patient^13^. Our study did not include a CRC or mismatch-repair-deficient patient who received COVID-19 vaccination. It is possible that the clinical course observed by Au et al. is a CRC or mismatch-repair-deficiency-specific effect given the distinct T-cell inhibitory mechanisms in these tumors, which may render T cells more responsive to PD-1 disinhibition^13,24^.

Notably, one patient in our study experienced grade 2 CRS before any COVID-19 vaccination was administered, highlighting that CRS can occur independently of vaccination under ICI therapy and may not necessarily be vaccine-related. This is particularly important in patients with cancer in a palliative setting with limited treatment options, as CRS treatments such as glucocorticoids may impair ICI efficacy and deprive patients of an important treatment option^25,26^. Our results suggest that CRS-related cytokines are commonly induced after COVID-19 vaccination and are not sufficient to establish the diagnosis of CRS. Clinically relevant CRS should therefore be diagnosed in symptomatic patients after an exhaustive differential diagnosis.

Two cases of severe COVID-19 (95% CI 1.6–22.5%) occurred in our unvaccinated cohort but none in our vaccinated patients. We observed induction of neutralizing antibodies after vaccination, thus corroborating current evidence that COVID-19 vaccines may have meaningful activity in ICI-treated patients with cancer^8^.

Vaccinated patients also showed increased overall survival in our study and vaccination status was an independent predictor of overall survival in a Cox proportional hazards model. A similar observation was reported in influenza-vaccinated patients with cancer who showed prolonged overall survival under ICI compared to unvaccinated patients^27^. In our study, this result was further supported by landmark analysis making guarantee-time bias an unlikely but not impossible explanation for this observation. Moreover, the prolonged overall survival in vaccinated patients cannot be explained by COVID-19-related mortality of unvaccinated patients alone. In unvaccinated patients only one in nine deaths was caused directly by COVID-19 infection and one death occurred due to tumor progression shortly after COVID-19 infection. As patients underwent regular rapid antigen-testing (q1w-q4w) it is unlikely that we missed a relevant number of COVID-19 cases. It is possible that the small sample size of our heterogenous cohort may have skewed the survival analysis despite the similarity of vaccinated and unvaccinated patients in many clinical covariates. Our results should therefore be validated in larger patient cohorts. Alternatively, patients with worse disease status and symptoms may be more hesitant and less likely to get vaccinated. This hypothesis is supported by the fact that unvaccinated patients experienced numerically more severe AEs. This observation may also be a result of increased health awareness or higher compliance regarding oncological therapy in vaccinated patients, an outcome that we did not assess in this study. Another possible explanation is that the cytokine boost induced by vaccination may have reinforced antitumor immunity. Specifically, IL-2 induction, as observed in our vaccinated patients, can break ICI resistance in subcutaneous murine models^28^. Despite the favorable survival data for our vaccinated patients, it should be noted that two patients died within 4 weeks after vaccination. It seems unlikely that these were causally related to the vaccine given plausible alternative explanations, namely computed tomography-confirmed hepatic tumor progression in one patient and severe aortic stenosis in combination with abrupt discontinuation of all cardiac medication by the other patient. However, cardiovascular events after COVID-19 vaccinations have been reported and should be closely monitored^29^. Overall, the tested COVID-19 vaccines were linked to favorable outcomes in our study, may have meaningful clinical activity in ICI-treated patients with cancer and warrant validation in larger ICI-treated patient cohorts.

Despite these insights, our study also has several limitations which should be considered in its interpretation. This is a cohort study and patients were not randomly assigned to vaccination. This led to a difference in AE propensity before vaccination as compared to unvaccinated patients, which given the observational nature of the study might have biased assessment of post-vaccination AEs. AEs and cytokine levels under SARS-CoV-2 vaccination were not the primary end point of this study; therefore, sample size was not optimized for these end points and our trial was not powered to estimate the exact frequency of rare AEs under ICI therapy and COVID-19 vaccination. Larger studies are necessary to determine this, although even phase 3 trials are often not powered for this comparison^30^. Moreover, AEs were assessed upon presentation at our day clinic every 1–6 weeks and not at a standardized early time point, as performed for randomized controlled vaccination trials^9^. While serious AEs were generally reported instantly, lower grade AEs may be underreported due to recall bias. Finally, all serum cytokine and antibody titer analyses were research grade and absolute concentrations from this study should not be used to establish clinical diagnoses. It is important to note that these drawbacks, such as small sample size and lack of randomization, also apply to other studies that have analyzed COVID-19 vaccinations in patients with cancer^8,14,15^. Strengths of our analysis include the prospective design, prospective recruitment of most patients, long-term follow-up, broad array of cancer types and combination immune-checkpoint therapies.

In summary, our data indicate that induction of CRS-related cytokines after COVID-19 vaccination is common in ICI-treated patients with cancer, but is generally not associated with clinical CRS symptoms meeting current CRS diagnostic criteria. Hence, cytokine induction is not sufficient to diagnose CRS in these patients. Our data warrant validation in larger cohorts to define the exact frequencies of CRS and other AEs in ICI-treated patients with cancer receiving COVID-19 vaccination. Overall, our study supports current clinical practice of COVID-19 vaccination in patients with cancer under ICI therapy.

## Methods

### Clinical trial

The presented trial was conducted in accordance with the declaration of Helsinki in its current edition and all relevant ethical regulations. The trial received institutional ethics review board approval at Ethics Commission I Medical Faculty Heidelberg, Heidelberg University (S-373/2020, S-207/2005) and Ethics Commission II Medical Faculty Mannheim, Heidelberg University (2021–567). Trial personnel were subject to medical confidentiality (paragraph 9 (Muster-)Berufsordnung für die in Deutschland tätigen Ärztinnen und Ärzte), the General Data Protection Regulations (DSGVO) and the Data Protection Act of the state of Baden-Württemberg (LSDG).

Patients presented in this study are part of the exploration cohort of a prospectively registered cohort study (DRKS00022890). The exploration cohort consisted of 220 patients of whom 166 were recruited prospectively and 54 retrospectively. Of these patients, 29 patients with dermatological cancers were also included in a retrospective survey study at our center^31^. All these patients are part of the here-reported prospectively registered trial and gave written informed consent for the investigations. For prospectively recruited patients this consent was study specific and for retrospectively recruited patients the consent was general but included the here-reported investigations. The trial including both prospective and retrospective patients has been approved by the Ethics Commission I Medical Faculty Heidelberg, Heidelberg University (S-373/2020 and S-207/2005) and Ethics Commission II Medical Faculty Mannheim, Heidelberg University (2021–567). Patients consented for data to be stored for up to 10 years after trial completion by Heidelberg University Hospital. All patients had been provided with contact numbers to request data deletion and their rights within the DSGVO as well as contact information of relevant oversight authorities. None of the here-reported patients requested data deletion, modification or restriction of its use and no patient withdrew consent. Patients consented that their data may be published in pseudonymized form and consented that after publication, deletion of the published data and analyses is no longer possible.

Adult patients with advanced solid tumors starting a new cancer immunotherapy either as mono- or combination therapy, excluding adoptive cell therapies were eligible for inclusion. Written informed consent, hemoglobin levels ≥80 g l^−1^ when additional blood samples were obtained and measurable disease according to RECIST 1.1 were obligatory requirements for inclusion. Patients received no compensation for participating in this trial. This is a single-arm cohort study and hence no randomization was performed. Patients were followed up at least every 1–6 weeks depending on the treatment regimen. AEs were continuously retrieved from electronic patient health records and graded according to CTCAE v.5.0 as per the trial protocol (German Clinical Trial Register DRKS00022890). Additionally, we applied the ASTCT 2019 criteria for CRS, which are more specific to immunotherapies (developed for adoptive cell therapies) but otherwise are very similar to CTCAE v.5.0. In contrast to previous CTCAE criteria neither the CTCAE v.5.0 nor the ASTCT criteria include organ toxicities in CRS diagnosis. The CTCAE v.5.0 and the ASTCT 2019 criteria require fever, hypoxia or hypotension as the defining feature of clinical CRS^11^. These criteria are likely imperfect and may require adaptation in the future. One example is that fever is also sufficient to diagnose CRS grade I according to these criteria but as noted by June and Fajgenbaum^12^ is also part of many appropriate clinical reactions that should not be diagnosed as CRS. We therefore only considered CRS grade ≥II as clinically relevant.

Other metadata collected included age at time of written informed consent, sex, tumor type, stage and histology, mutational status, sites of metastases, history of tobacco use, pre-existing health conditions, concurrent medication and survival. Pre-existing health conditions were obtained from electronic patient records and defined according to the Side Effect Resource (SIDER v.4.1: http://sideeffects.embl.de). Every week to every four weeks, patients also received regular clinical laboratory tests, including creatinine, bilirubin, CRP, hemoglobin, platelet and leukocyte counts. Additionally, patients received longitudinal blood samples for cytokine measurements before the start of therapy, within 1–7 weeks after therapy initiation and every 8–12 weeks under immunotherapy. COVID-19 vaccination status was assessed during regular follow-up. Patients did not undergo any additional screening for determination of vaccination status.

The primary outcome measure of the trial was prediction of radiological response, which will be reported elsewhere. Secondary outcome measures included the serum proteome and peripheral blood immune cell composition overall, grade 3 AEs as well as progression-free and overall survival. Patient health information is pseudonymized.

### Analysis of serum cytokines and neutralizing antibodies

Blood was collected either peripherally through venipuncture or via a central port catheter in coagulation matrix containing serum tubes (no. 01.1602, Sarstedt) from live patients with cancer. Clinical characteristics and tumor types are indicated in Table 1. Samples were kept at room temperature until preparation (generally <6 h, but always <24 h). Samples were prepared at the National Center for Tumor Diseases (NCT) Liquidbank biobank using a standard operating procedure: for serum preparation, tubes were centrifuged at 2,500*g* for 10 min at room temperature and the upper phase was transferred into 500-µl aliquots and stored at −80 °C. For both cytokine and antibody analysis we only selected patients with baseline samples obtained within 6 months before vaccination. For cytokine analysis we selected all samples obtained up to 6 weeks after vaccination. For antibody analysis we selected all samples until the end of follow-up. Samples were analyzed at NCT Heidelberg or the German Cancer Research Center (DKFZ) immediately after thawing; no shipping was required. Reporting was conducted in accordance with BRISQ criteria^32^.

Serum samples were thawed and immediately analyzed in duplicate using the Legendplex Cytokine Storm Panel 1 (AB_2895549 (antibodyregistry.org), 741091, BioLegend), Cytokine Storm Panel 2 (AB_2895550 (antibodyregistry.org), 741142, BioLegend) or SARS-CoV-2 Neutralizing Antibody Assay (AB_2895551 (antibodyregistry.org), 741127, BioLegend) according to manufacturer’s instructions and analyzed on a BD FACS Canto II flow cytometer (BD) using the BD FACS DIVA v.8.0 software (BD). In brief, patient serum was diluted to 1:2 or to 1:100 of the initial concentration for the cytokine multiplex and neutralizing antibody assay, respectively. Diluted serum, standard, assay buffer, matrix, antibody-coupled capture beads and biotinylated detection antibodies were transferred to a 96-well V-bottom microplate. After incubation and washing streptavidin-PE was added to each well and cells were transferred to flow cytometry tubes and acquired using a FACS Canto II (BD). The gating for flow cytometry analysis is depicted in Extended Data Fig. 1. Analyte concentrations were interpolated from a standard curve using 5 parameter logistic regression using Legendplex Software v.2021.07.01 (Biolegend). Cytokine concentrations below the lower limit of detection were set to 0.

### General data analysis

All data analysis was performed using Python 3 in a Jupyter notebook or GraphPad Prism v.9.2.0 (GraphPad Software). All computer code is provided under GitHub at https://github.com/wallet-maker/ANTICIPATE_COVID-19 and https://zenodo.org/record/6544522#.YoYKBXXMLcs. Plotting was conducted using the Matplotlib (v.3.4.3) and Seaborn (v.0.11.2) packages. Other package versions included mgaug (v.0.2.5), pandas (v.0.23.4), squarify (v.0.4.3), lifelines (v.0.26.4), statsmodels (v.0.10.2), numpy (v.1.19.5), mpmath (v.1.2.1), scipy (v.1.4.1), pymatch (v.0.3.4) and Jupyter (v.1.0.0). Plots were arranged using Adobe Illustrator 2021 (v.25.2.2, Adobe).

### Bootstrapping cytokine–cytokine clustering probabilities

We transformed cytokine concentrations according to the following formula *c*_t_ = log_10_(*c* + 1) with *c*_t_ as the log1p-transformed cytokine concentration and *c* as the raw cytokine concentration in pg ml^−1^ for all vaccinated patients. We then normalized all post-vaccination log1p-transformed concentrations by subtracting the respective log1p-transformed cytokine concentrations of the baseline sample. Based on these normalized concentrations, we then calculated a Pearson cytokine–cytokine correlation matrix. We then used the correlation distances as an input for the scipy.cluster.hierarchy.linkage function with the unweighted pair group method with arithmetic mean, to obtain the row and column linkages and transformed these into flat clusters by applying scipy.cluster.hierarchy.fcluster function using a cophenetic distance of 0.75.

We sampled the normalized log1p-transformed cytokine concentration dataframe object with replacement with the same sample size as the initial dataframe and repeated the above-mentioned procedure to obtain flat clusters. This sampling and clustering was repeated *n* = 10,000 times. For each pair of cytokines we then counted their co-occurrence in a cluster and summed the values for all 10,000 separate clusterings dividing the counts for each pair of cytokines by 10,000 to obtain an approximation of the probability for each pair of cytokines to fall into the same cluster.

### Time-to-event analysis

Survival time or time to first overall or first CTCAE v.5.0 grade ≥3 adverse event was analyzed by Kaplan–Meier curves and log-rank tests (Mantel–Cox) using GraphPad Prism and the lifelines package (v.0.26.4). A Cox proportional hazards model was calculated using the lifelines package. We inspected the Kaplan–Meier curves and did not see any obvious violation of the proportional hazards assumption; however, when formally testing the proportional hazards assumption using the check_assumptions function in the lifelines packages the ‘age’ variable violated this assumption when used on a continuous level. We therefore stratified patients into >60 and ≤60 years of age at inclusion. To gauge the effect of vaccination on AEs we calculated Kaplan–Meier statistics and event rates (events per days at risk) for different intervals: over the entire observation period (for vaccinated and unvaccinated patients), for vaccinated patients before vaccination, for the first 28 d after the first vaccination dose and for >28 d after the first vaccination dose until the end of follow-up. We did not perform time-to-event analysis for irAEs because of the limited power of this analysis. Using the lifelines package, we also calculated a Cox proportional hazards model using the variables indicated in Fig. 4d. When no events were observed in one group we reported hazard ratios using the Mantel–Hanszel method implemented in GraphPad Prism v.9.2.0. Otherwise, we used the log-rank method implemented in GraphPad Prism v.9.2.0.

### Landmark analysis

We performed a landmark analysis for overall survival before or after the landmark of all vaccinated and unvaccinated patients. The landmark was set as 17 May 2020, which was the date of general eligibility for COVID-19 vaccination in the state of Baden-Württemberg, Germany (press release of the Ministry of Social Affairs, Health and Integration of the state of Baden-Württemberg, Germany, 12th May 2021: https://sozialministerium.baden-wuerttemberg.de/de/service/presse/pressemitteilung/pid/priorisierung-in-arztpraxen-ab-17-mai-fuer-alle-impfstoffe-aufgehoben/). Patients with cancer had prioritized access to COVID-19 vaccines before this landmark and consequently 91.9% of all patients vaccinated at some point in this study were vaccinated until this date. To assess overall survival after the landmark, patients not vaccinated up to that date were counted as unvaccinated as required in landmark analysis. *P* values were calculated using log-rank tests.

### Propensity score matching

Propensity score matching was performed using the pymatch package (v.0.3.4) and the exact procedure is outlined in the publication describing the package^33^. In brief, a linear regression model is fitted using either ECOG ∈ {≤1,>1}, age ∈ positive integers and sex ∈ {male, female} or private insurance status ∈ {true, false}, age ∈ positive integers and sex ∈ {male, female} as *k* = 3 independent variables per model. The propensity scores are defined as π_i_ = π (X_i_) = Pr(*T*_i_ = 1 | *X*_i_) with *X*_i_ = (*X*_i1_, *X*_i2_, *X*_i3_, … *X*_ik_) being a vector of *k* features for each patient i and *T*_i_ the class membership of each patient with *T*_i_ = 1 if the patient was vaccinated and *T*_i_ = 0 if the patient was unvaccinated. The logistic regression model was defined as log(π_i_ / (1 − π_i_) = *X*_i_ β + ε_i_, i = 1,2,…, *n*.

Based on these propensity scores we assigned each unvaccinated patient a vaccinated counterpart with replacement. This led to partly efficient matching, defined as a reduction in the age and sex imbalance of the vaccinated and unvaccinated cohorts (Extended Data Figs. 5b and 6b). We did not include other variables in the calculation of propensity score because they led to perfect separation. We then compared AEs occurring under ICI therapy in unvaccinated and matched vaccinated patients.

### Statistics and reproducibility

The study size was defined by sample size estimation based on the primary outcome parameter (radiological response) as outlined in the study protocol. Briefly, an area under the curve (AUC) >0.78 for predicting radiological response was chosen as a clinically relevant threshold based on current literature. We used published data to model a receiver operator curve (sensitivity versus 1 − specificity) and based on this, calculated AUC confidence intervals for different patient numbers assuming a response rate of 10%. A patient number of *n* = 200 resulted in a lower limit of AUC = 0.782 we estimated a dropout of 10%, resulting in a total of 220 patients for the training and 220 patients for the testing cohort. For the here-reported exploratory study outcomes, no statistical method was used to predetermine sample size. We excluded six patients who were fully vaccinated before the start of immunotherapy because these patients would have confounded the interpretation of study results. For the cytokine analysis, we excluded one additional patient who had an irAE at baseline before vaccination. Cytokine concentrations were measured in technical duplicate in three independent experiments with different patient subgroups (outlined in the provided code) with similar results for all these subgroups. Antibody concentrations were measured in one experiment in technical duplicate. The experiments were not randomized. Investigators retrieving patient metadata and outcome assessments were not blinded to vaccination status. The acquisition and processing of the raw cytometry data was performed by a clinician scientist who was blinded to the patients’ identity and metadata and who was not involved in downstream data analysis; however, the patients’ pseudonyms contained the temporal sequence of the samples.

Confidence intervals for frequencies/proportions were calculated as Clopper–Pearson intervals based on the β distribution using the statsmodels.stats.proportion.proportion_confint function of the statsmodels package (v.0.10.2). *P* values were calculated using Wilcoxon one-sample tests or Wilcoxon matched-pairs signed-rank tests for continuous/ordinal one-sample or paired two-sample data, respectively using scipy’s scipy.stats.wilcoxon function (v.1.7.2). There was no indication for violating the assumptions of these nonparametric tests such as asymmetric difference scores. Proportional data were analyzed using a chi-squared test because its assumptions were met (two or more categorical independent variables). The Fisher’s exact test could not be used because the number of patients experiencing an event (such as an AE) was unconditioned. Survival data were analyzed using log-rank tests or Cox proportional hazards models. There was no indication that the proportional hazards assumption was violated, such as crossing of the Kaplan–Meier curves. We formally tested the proportional hazards assumption using the check_assumptions function of the lifelines Python package, which showed no indication of violation after stratifying the age variable. All *P* values are two-tailed. For cytokine data analysis *P* values were corrected for multiple comparisons with the Benjamini–Hochberg method using R v.4.1.1 and the p.adjust function. For clinical data analysis we did not use multiple comparisons correction to increase our power to detect differences in vaccine-related AEs.

### Reporting summary

Further information on research design is available in the Nature Research Reporting Summary linked to this article.

## Supplementary information

Supplementary Information Supplementary Note and study protocol.

Reporting Summary

Supplementary Table Supplementary Tables 1–6.

## Data Availability

Data on cytokine serum concentrations and patient metadata generated for this study are publicly available through Zenodo (https://zenodo.org/record/6544522#.YoYUQXXMLcs). Patient data will be anonymized 10 years after study completion. Source data for all figures are provided as Source Data files. All other data supporting the findings of this study are available from the corresponding author on reasonable request. Source data are provided with this paper.

## Source data

Source Data Fig. 1 Statistical Source Data.
Source Data Fig. 2 Statistical Source Data.
Source Data Fig. 3 Statistical Source Data.
Source Data Fig. 4 Statistical Source Data.
Source Data Extended Data Fig. 2 Statistical Source Data.
Source Data Extended Data Fig. 3 Statistical Source Data.
Source Data Extended Data Fig. 4 Statistical Source Data.
Source Data Extended Data Fig. 5 Statistical Source Data.
Source Data Extended Data Fig. 6 Statistical Source Data.
Source Data Extended Data Fig. 7 Statistical Source Data.
Source Data Extended Data Fig. 8 Statistical Source Data.
Source Data Extended Data Fig. 9 Statistical Source Data.
Source Data Extended Data Fig. 10 Statistical Source Data.

## Code availability

All custom computer code used to generate the results in this study has been deposited in a GitHub repository (https://github.com/wallet-maker/ANTICIPATE_COVID-19.git) and a Zenodo repository (https://zenodo.org/record/6544522#.YoYUQXXMLcs)^34^ under an MIT license.
