# Supplementary material for: Cytokine release syndrome-like serum responses after COVID-19 vaccination are frequent and clinically inapparent under cancer immunotherapy
Source: Nat Cancer. 2022 Jun 17;3(9):1039–51. doi: 10.1038/s43018-022-00398-7 (PMC9499865; doi:10.1038/s43018-022-00398-7)
Supplement: Supplementary file 1 — Supplementary Note and study protocol. [file 43018_2022_398_MOESM1_ESM.pdf]

nature portfolio

https://doi.org/10.1038/s43018-022-00398-7

Supplementary information

Cytokine release syndrome-like serum responses after COVID-19 vaccination are frequent and clinically inapparent under cancer immunotherapy

In the format provided by the authors and unedited

Cytokine release syndrome-like serum responses after COVID-19 vaccination are frequent but clinically inapparent in cancer patients under immune checkpoint therapy

Thomas Walle[1,4,5,6*,#], Sunanjay Bajaj[1,5,6,#], Joscha A. Kraske[5,6], Thomas Rösner[5,6], Christiane S. Cussigh[7], Katharina A. Kälber[7], Lisa Jasmin Müller[8], Sophia Boyoung Strobel[7], Jana Burghaus[7], Stefan Kallenberger[5,6], Christoph Stein-Thöringer[2,5,6], Maximilian Jenzer[5,6], Antonia Schubert[,4,5,6,13,14], Steffen Kahle[5,6], Anja Williams[5,6], Birgit Hoyler[1], Lin Zielske[10], Renate Skatula[10], Stefanie Sawall[1], Mathias F. Leber[1,5,6], Russell Z. Kunes[11], Johannes Krisam[12], Carlo Fremd[5,6,9], Andreas Schneeweiss[9], Jürgen Krauss[5,6], Leonidas Apostolidis[5,6], Anne Katrin Berger[5,6], Georg M. Haag[5,6], Stefanie Zschäbitz[5,6], Niels Halama[3,5,6], Christoph Springfeld[5,6], Romy Kirsten[10], Jessica C. Hassel[7], Dirk Jäger[5,], NCT ANTICIPATE Investigators & Guy Ungerechts[1,4,5,6,13]

Supplementary Information

This file contains the following supplementary data:
Supplementary Note with Supplementary Suppl. Note Fig 1-6
Study Protocol

Supplementary Tables S1-6 are submitted in a separate Microsoft Excel (.xlsx) file

Source data are supplied as separate Microsoft Excel (.xlsx) files:

- Figure 1: NATCANCER-A05726-T_Source_Data_Figure1.xlsx
- Figure 2: NATCANCER-A05726-T_Source_Data_Figure2.xlsx
- Figure 3: NATCANCER-A05726-T_Source_Data_Figure3.xlsx
- Figure 4: NATCANCER-A05726-T_Source_Data_Figure4.xlsx
- Figure ED1: NATCANCER-A05726-T_Source_Data_FigureED1.xlsx
- Figure ED2: NATCANCER-A05726-T_Source_Data_FigureED2.xlsx
- Figure ED3: NATCANCER-A05726-T_Source_Data_FigureED3.xlsx
- Figure ED4: NATCANCER-A05726-T_Source_Data_FigureED4.xlsx
- Figure ED5: NATCANCER-A05726-T_Source_Data_FigureED5.xlsx
- Figure ED6: NATCANCER-A05726-T_Source_Data_FigureED6.xlsx
- Figure ED7: NATCANCER-A05726-T_Source_Data_FigureED7.xlsx
- Figure ED8: NATCANCER-A05726-T_Source_Data_FigureED8.xlsx
- Figure ED9: NATCANCER-A05726-T_Source_Data_FigureED9.xlsx
- Figure ED10: NATCANCER-A05726-T_Source_Data_FigureED10.xlsx

Supplementary Note

The supplementary note contains a detailed history of all patients who were vaccinated and had post-vaccine AE that led to hospitalization and/or death. Each patient’s history is depicted in a timeline, and we discuss the beginning of immunotherapy, the various AE that we observed during therapy, the vaccination dates, the post-vaccine AE and the reasons for hospitalization and/or death in detail. All information is also available in the Supplementary Tables listing basic patient characteristics (Table S1), the AE observed (Table S2-3) and the reasons for hospitalizations (Table S4) and deaths (Table S5).

This concerns the following patients:
Patient 32PHU4
Patient QH6V9I
Patient QLWN64
Patient SVXZIG
Patient OI9CSG
Patient 7Z9V9Q

Abbreviations: ICI - immune checkpoint inhibitor therapy;

Patient 32PHU4

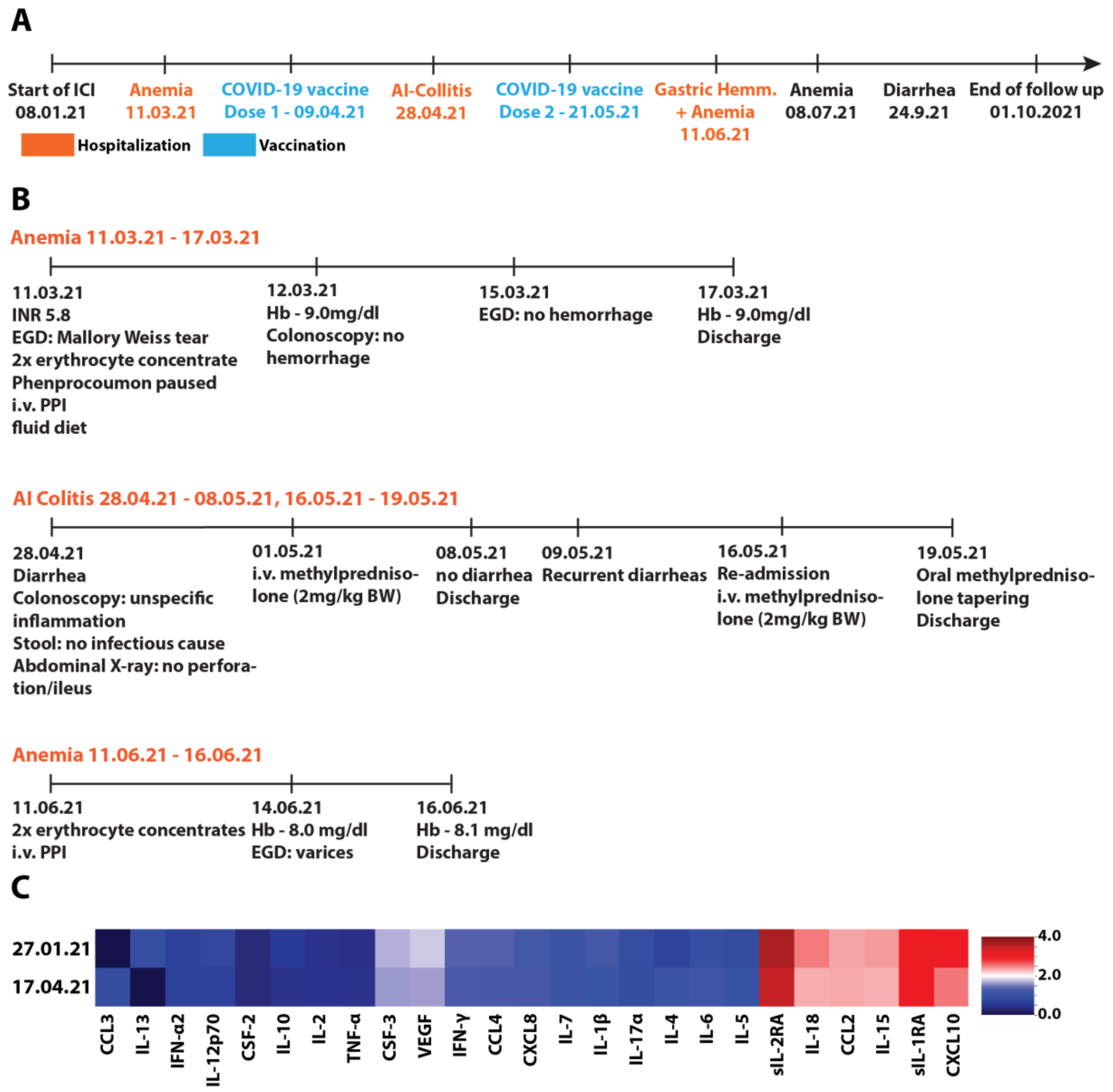

Suppl. Note Fig 1 Post-vaccination serious adverse events and cytokine release in patient 32PHU4
(A) Timeline indicating adverse events, vaccinations (in blue) and hospitalizations (adverse events highlighted in orange).
(B) Timelines depicting the clinical course of the patient in the indicated periods of hospitalization
(C) Heatmap showing the $_{10}$log1p transformed cytokine concentrations before vaccination and after the first vaccination dose with sampling dates indicated on the left. We observed no relevant increase in serum cytokine levels after the first vaccination (17.04.21 9d after 1st vaccination) as compared to pre-vaccination levels (27.1.2021).
Abbreviations: AI: autoimmune, BW: body weight; Hb: hemoglobin; ICI: immune checkpoint therapy; i.v.: intravenous, freq.: frequency, PPI: proton pump inhibitor.

**Tumor:**	Hepatocellular Carcinoma
**ICI:**	Ipilimumab + Nivolumab
**Therapy line:**	3rd line systemic oncological therapy
**Start of ICI:**	08.01.21

Comorbidities: Arterial Hypertension, Liver Fibrosis, Type 2 Diabetes, Aortic Valve Implantation, Hyperuricemia, Lower Leg Amputation (Left), Gastritis, Pancreatic IPMN

Covid-19 vaccination:
1st dose: 09.04.21 BNT162b2
2nd dose: 21.05.21 BNT162b2

Survival: Patient alive as of 1.10.2021

73-year old male patient who started immunotherapy with Ipilimumab and Nivolumab as a 3rd line therapy for irresectable hepatocellular carcinoma with liver fibrosis on 08.01.2021 (**Figure T1A**). Liver fibrosis was most likely caused by cardiac disfunction (cirrhosis cardiaque). After initially tolerating the therapy well, he was hospitalized in an external hospital from 11.03.21-17.03.21 with grade 4 anemia due to upper gastrointestinal hemorrhage under phenprocoumon anticoagulation with international normalized ratio (INR) coagulation value derangement (INR 5.8) (**Figure T1B**). An esophago-gastroduodenoscopy on 11.03.21 revealed upper gastrointestinal hemorrhage from a Mallory-Weiss lesion >2cm without active bleeding. The patient received two erythrocyte concentrates, was switched to fluid diet and i.v. proton pump inhibitor therapy was initiated. INR values normalized after pausing phenprocoumon. Because of a hyperthyroidism which was attributed to the CT radiocontrast agent he received a single dose of carbimazol which he did not tolerate well, and which led to hypothyroidism. He was discharged in stable condition and normalized improved hemoglobin of 9.0mg/dl on 17.03.21 under continued anticoagulation with phenprocoumon. Hypothyroidism persisted for many months after discharge and was treated with oral l-thyroxine.

The patient received his 1st COVID-19 vaccination dose on 09.04.21 and reported no immediate adverse events. We noticed no relevant increase in serum cytokine levels after the first vaccination as compared to pre-vaccination levels (**Figure T1C**). We continued immune checkpoint therapy on 16.04.21 and after an initially stable condition the patient reported watery diarrheas on 28.04.21 and was hospitalized for in-patient therapy and diagnostics. The patient reported no fever, stool samples found no evidence for Clostridium difficile and sigmoidoscopy biopsies showed unspecific signs of inflammation leading to a diagnosis of ICI induced colitis. The patient received 2mg/kg body weight methylprednisolone as per European Society of Medical Oncology (ESMO) guidelines (*1*) under which diarrheas initially ceased and the patient was discharged on oral glucocorticoids on 08.05.21. However, diarrheas increased in frequency under oral glucocorticoids and the patient was re-admitted on 16.05.21. The patient did not experience fever and i.v. methylprednisolone was reinitiated leading to cessation of diarrheas. On 19.05.21 we discharged the patient under oral glucocorticoid tapering. Shortly after being discharged, the patient received his 2nd COVID-19 vaccination dose on 21.5.2021 which was tolerated well and did not lead to recurrence of diarrheas.

However, we noted grade 4 anemia on 11.06.21 with severe fatigue and general weakness and again admitted the patient to our inpatient ward (**Figure T1B**). Methylprednisolone dose at that time was 2.5mg/day. We transfused 2 erythrocyte concentrates and admitted the patient to our inpatient ward. Esophago-gastroduodenoscopy revealed upper gastrointestinal hemorrhage from a variceal lesion <1cm without active hemorrhage. On 14.6.21 the patient developed mild fevers of 38.6°C which responded to ampicillin/sulbactam antibiotic treatment. We chose to not increase glucocorticoid therapy and discharged the patient under oral antibiotics. After continuation of ICI on 25.06.21 the patient once again experienced grade 3 anemia and we transfused erythrocyte concentrates in our day clinic. After stabilization of blood hemoglobin values the patient continued therapy on 16.07.21. Shortly before the end of our observation period on 1.10.2021, diarrheas reoccurred on 24.09.21 (not shown on timeline) therapy was discontinued and methylprednisolone 2mg/kg body weight was reinitiated under which diarrheas ceased.

Patient QH6V9I

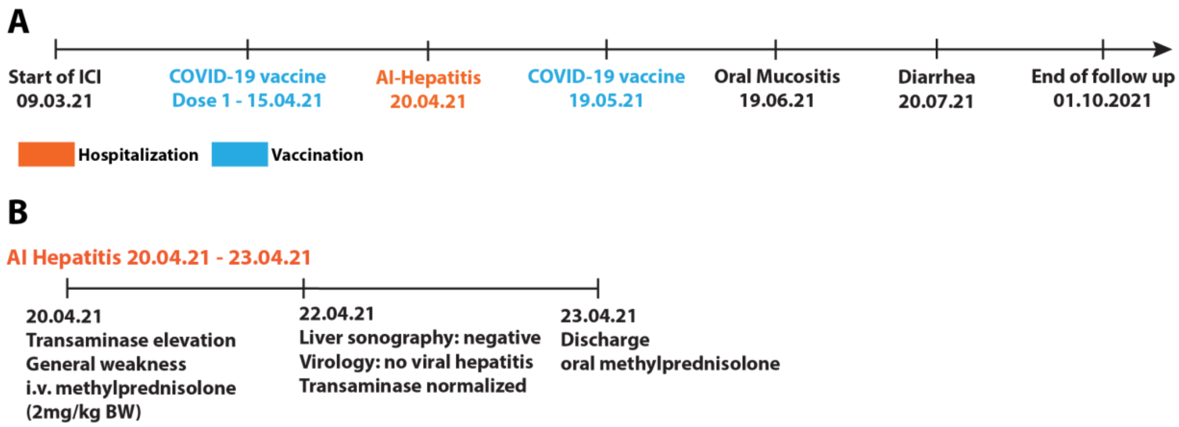

Suppl. Note Fig 2 Post-vaccination serious adverse events in patient QLWN64
(A) Timeline indicating adverse events, vaccinations (in blue) and hospitalizations (adverse events highlighted in orange).
(B) Timelines depicting the clinical course of the patient in the indicated periods of hospitalization
Abbreviations: AI: autoimmune, C.diff: Clostridium difficile; ICI: immune checkpoint therapy; i.v.: intravenous, freq.: frequency.

**Tumor:**	Clear cell renal carcinoma
**ICI:**	Pembrolizumab + Axitinib
**Therapy line**:	1st line systemic oncological therapy
**Start of ICI**:	09.03.21
**Comorbidities**:	Arterial Hypertension, Hypercholesterolemia, Benign Prostate Syndrome, Renal Cyst

Covid-19 vaccination:

**1st dose:**	15.04.21 mRNA-1273
**2nd dose:**	19.05.21 mRNA-1273

**Survival:**	Patient alive as of 1.10.2021

67-year-old male patient who began 1st line therapy with Pembrolizumab and Axitinib for metastatic clear-cell renal cell carcinoma on 09.03.21 (**Figure T2A**). He received the first two cycles without major adverse events and received the first COVID-19 vaccination on 15.04.21. On 20.04.21 he experienced muscular weakness, fatigue and a sudden increase of liver transaminases and was hospitalized due to suspected grade 3 autoimmune hepatitis under ICI (**Figure T2B**). We paused axitinib and pembrolizumab and initialized 2mg/kg body weight i.v. methylprednisolone therapy under which liver transaminases normalized and we discharged the patient on 23.04.21 in stable condition. The patient received his 2nd COVID-19 vaccination dose without AEs within 4 weeks after vaccination. We reinitiated axitinib therapy on 11.5.21. The patient subsequently experienced oral and nasal mucositis on 19.06.21. Once mucositis subsided and patient was in stable condition, and we resumed pembrolizumab and axitinib at reduced dose (3mg bi-daily) on 29.06.21. However, he experienced °III diarrheas from 20.07.21 which were attributed to axitinib and improved after reduction to a dose of 2mg bi-daily but intermittently persisted until the end of follow-up on 01.10.21.

Patient QLWN64

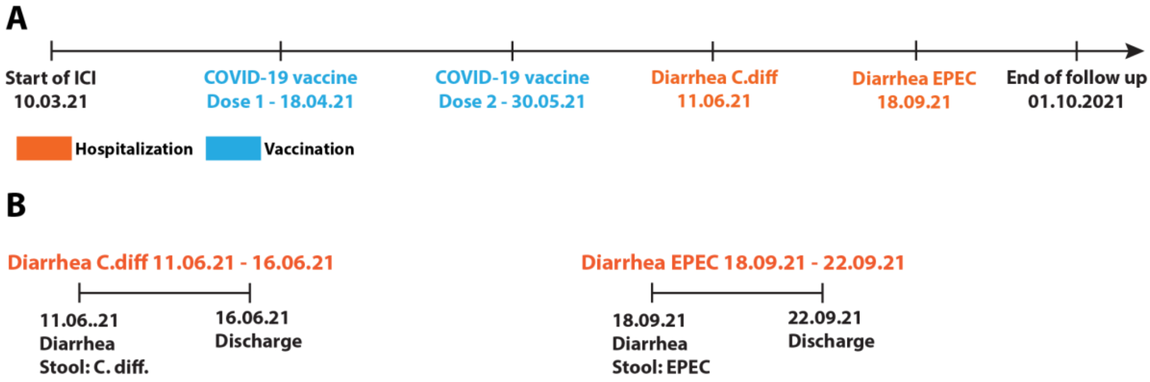

Suppl. Note Fig 3 Post-vaccination serious adverse events in patient QLWN64
(A) Timeline indicating adverse events, vaccinations (in blue) and hospitalizations (adverse events highlighted in orange).
(B) Timelines depicting the clinical course of the patient in the indicated periods of hospitalization
Abbreviations: AI: autoimmune, C.diff: Clostridium difficile; ICI: immune checkpoint therapy; i.v.: intravenous, freq.: frequency.

**Tumor:**	Clear cell renal carcinoma
**ICI:**	Pembrolizumab + Axitinib
**Therapy line**:	1st line systemic oncological therapy
**Start of ICI**:	10.03.21
**Comorbidities**:	Arterial Hypertension

Covid-19 vaccination:

**1st dose:**	18.04.21 BNT162b2
**2nd dose:**	30.05.21 BNT162b2

**Survival:**	Patient alive as of 1.10.2021

52 year-old male patient who began 1st line therapy with pembrolizumab and axitinib for metastatic clear-cell renal cell carcinoma on 10.03.21 (**Figure T3A**). He initially tolerated therapy well without apparent adverse events and received the BNT162b2 vaccine on 18.04.21 after which no adverse events occurred. He received the second BNT162b2 dose on 30.05.21. On 11.6.21 the patient was admitted due to grade 3 diarrhea clostridium difficile infection was diagnosed and after antibiotic treatment the patient was discharged on 11.06.21 (**Figure T3B**). On 18.9.21 the patient was again admitted for diarrhea due to enteropathogenic E. coli (EPEC) colitis grade 3 (**Figure T3B**). He was discharged on 22.9.21 but diarrhea quickly reoccurred upon axitinib therapy. Consequently, axitinib therapy was reduced to 3mg bi-daily under which diarrheas ceased completely.

Patient SVXZIG

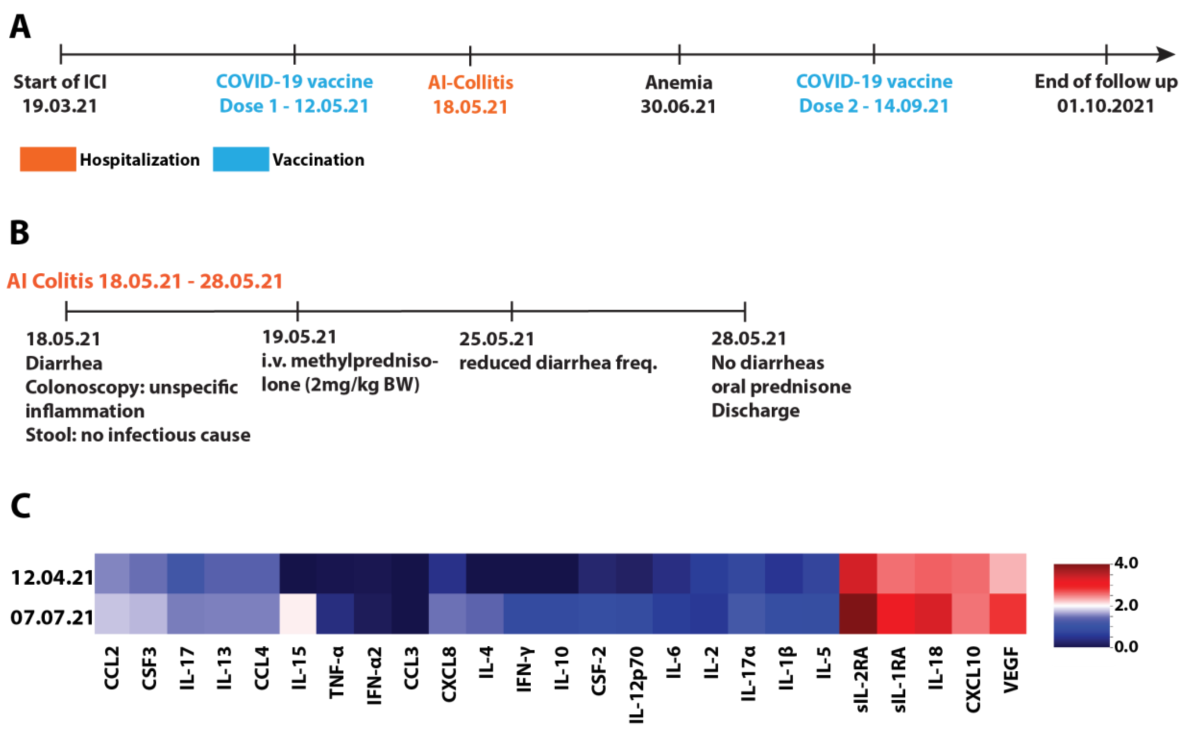

Suppl. Note Fig 4 Post-vaccination serious adverse events and cytokine serum concentrations in patient SVXZIG
(A) Timeline indicating adverse events, vaccinations (in blue) and hospitalizations (adverse events highlighted in orange).
(B) Timelines depicting the clinical course of the patient in the indicated periods of hospitalization
(C) Heatmap showing the $_{10}$log1p transformed cytokine concentrations before vaccination and after 50 days the first vaccination dose with sampling date indicated on the left. We observed high sIL-2RA levels before (12.04.21) and after (07.07.21, 56 days after 1st vaccination) the 1st vaccination.
Abbreviations: AI: autoimmune, ICI: immune checkpoint therapy; i.v.: intravenous, freq.: frequency.

**Tumor:**	Melanoma
**ICI:**	Ipilimumab + Nivolumab
**Therapy line:**	1st line systemic oncological therapy
**Start of ICI:**	19.03.21
**Comorbidities**:	Arterial Hypertension, Depression, Psychosis

Covid-19 vaccination:

**1st dose:**	12.05.21 BNT162b2
**2nd dose:**	21.06.21 BNT162b2

**Survival:**	Patient alive as of 1.10.2021

68-year old female patient who started ipilimumab and nivolumab as a 1st line therapy for metastatic melanoma on 19.03.21 (**Figure T4A**). She received her first dose of BNT162b2 on 12.05.21. On 18.05.21 she was admitted to an external hospital because of grade 4 diarrhea (**Figure T4B**). After no other cause could be established, autoimmune colitis was suspected and the patient received 2mg/kg body weight i.v. methylprednisolone under which diarrheas ceased and she was discharged on 28.5.21 under oral glucocorticoid tapering. ICI was paused and not reinitiated until the end of follow-up 1.10.21. Notably, this patient showed high sIL-2RA levels

before the vaccination and clinical onset of the colitis event (**Figure T4C**) suggesting that some subclinical inflammatory condition, such as colitis, may have existed before onset of clinical symptoms (**Figure T4C**). However, it is also possible that vaccination-induced cytokine release contributed to the colitis event. She received her second COVID-19 vaccination dose on 14.09.21 under 2.5mg/day prednisone without adverse events especially no renewed onset of diarrheas. Glucocorticoids were discontinued on 15.9.21.

Patient OI9CSG

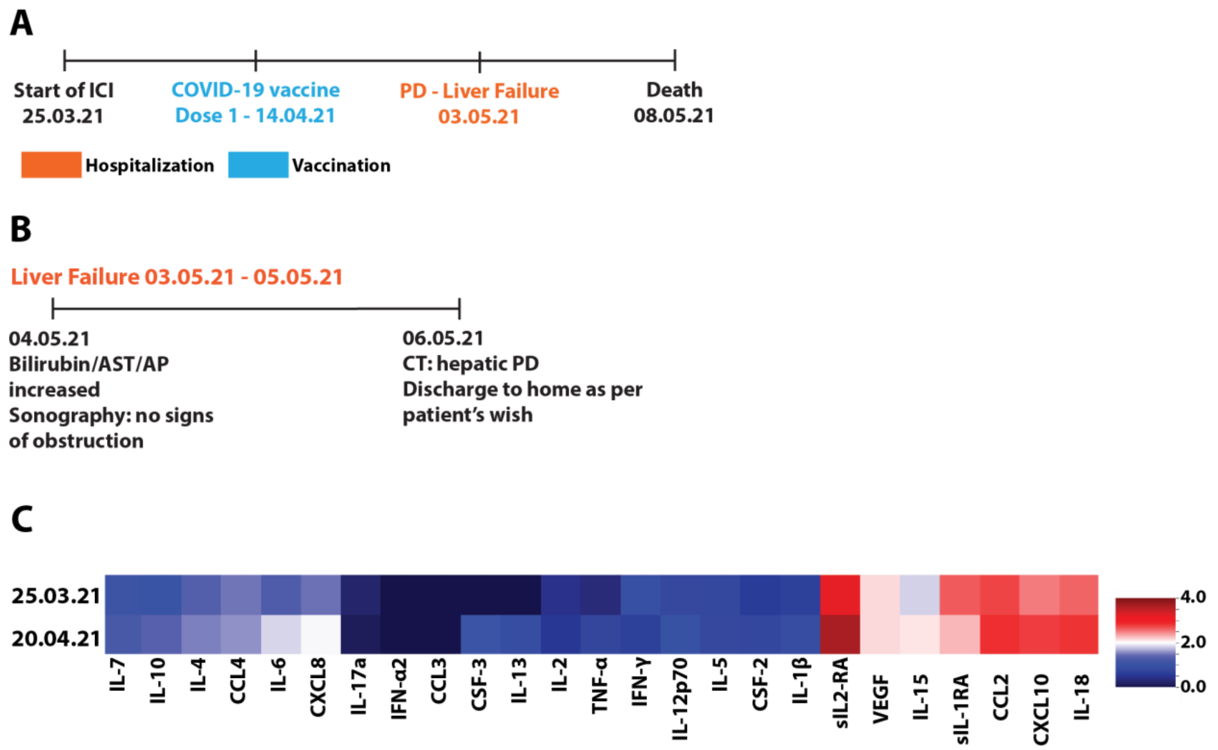

Suppl. Note Fig 5 Post-vaccination serious adverse events and cytokine serum concentrations in patient OI9CSG

(A) Timeline indicating adverse events, vaccinations (in blue) and hospitalizations (adverse events highlighted in orange).

(B) Timelines depicting the clinical course of the patient in the indicated periods of hospitalization

(C) Heatmap showing the $_{10}$log1p transformed cytokine concentrations before vaccination and after the first vaccination dose with sampling dates indicated on the left. We observed increased levels of several serum cytokines after the first COVID-19 vaccination (20.04.21, 6 days after the 1st vaccination).

**Tumor:**	Hepatocellular carcinoma
**ICI:**	Ipilimumab + Nivolumab
**Therapy line:**	1st line systemic oncological therapy
**Start of ICI:**	25.03.21
**Comorbidities:**	Arterial Hypertension, Hypercholesterolemia, Benign Prostate Syndrome, Macular Degeneration, Hypothyroidism

Covid-19 vaccination:

**1st dose:**	14.04.21 BNT162b2
**2nd dose:**	NA

**Survival:**	Patient died 8.5.21

80 year-old male patient who started ipilimumab and nivolumab as 1st line oncological systemic therapy for melanoma with hepatic, splenic, pulmonary, lymphatic and chest wall metastases on 25.3.21 (**Figure T5A**). Therapy was initially tolerated very well, and he received his first COVID-19 vaccination on 14.4.21. On 03.05.21, the patient was admitted to our inpatient ward because of

increasing fatigue and signs of cholestasis with darkening of urin and icteric sclerae (**Figure T5B**). Laboratory studies showed °II elevated bilirubin, °III alkaline phosphatase and °II aspartate transaminase elevation whereas alanine transaminase levels stayed within normal reference range indicating sever liver damage. Liver sonography showed no signs of obstruction. CT confirmed progressive disease with new and enlarged liver metastases. Given the rapidly deterioration of the patient he wished to not continue tumor-specific oncological therapy and was instead placed on best supportive care. We adjusted the patient's analgesia and nausea medication. The patient had no fever at any point during in-patient care. However, the patient showed increased levels of several serum cytokines after the first COVID-19 vaccination such as CXCL8, IL-6, CCL2, IL-18 and sIL-2RA (**Figure T5C**). On 06.05.21 we discharged to home as per his wish where he died on 08.05.21 due to hepatic failure most likely due to the CT confirmed hepatic tumor progression.

Patient 7Z9V9Q

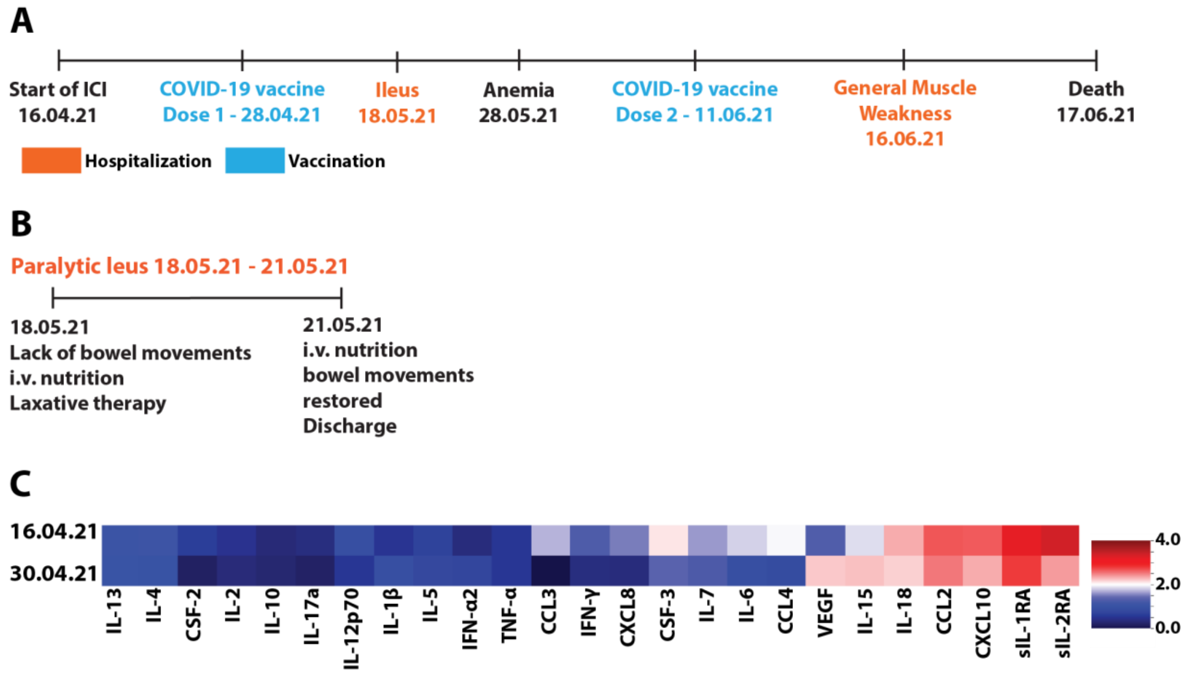

Suppl. Note Fig 6 Post-vaccination serious adverse events and cytokine serum concentrations in patient 7Z9V9Q
(A) Timeline indicating adverse events, vaccinations (in blue) and hospitalizations (adverse events highlighted in orange).
(B) Timelines depicting the clinical course of the patient in the indicated periods of hospitalization
(C) Heatmap showing the $_{10}$log1p transformed cytokine concentrations before vaccination and after the first vaccination dose with sampling dates indicated on the left.

**Tumor:**	Esophageal Squamous cell carcinoma, Lung Adenocarcinoma
**ICI:**	Nivolumab
**Therapy line**:	2nd line systemic oncological therapy
**Start of ICI**:	25.03.21
**Comorbidities**:	Arterial Hypertension, Hypercholesterolemia, Benign Prostate Syndrome, Macular Degeneration, Hypothyroidism

Covid-19 vaccination:

**1st dose:**	28.04.21 AZD1222
**2nd dose:**	09.06.21 AZD1222

**Survival:**	Patient died 17.06.21

76 year-old male patient who started nivolumab as a 2nd line therapy for concurrent metastatic lung adenocarcinoma and locally advanced squamous cell carcinoma of the esophagus (**Figure T6A**). He initially tolerated therapy well and received his 1st AZD1222 vaccination on 28.04.21. Most analyzed cytokine-release syndrome-associated serum cytokines decreased after vaccination (**Figure T5C**). On 18.05.21 he was hospitalized for lack of bowel movements for 6 days at an external hospital (**Figure T5B**). The patient was switched from percutaneous endoscopic gastroscopy tube feeding to i.v. nutrition *via* his port catheter. Bowel movements recurred after laxative measures and fluid management and he was discharged on 21.05.21. We restarted oncological therapy shortly thereafter and was well tolerated except for grade 2 anemia and a single episode of grade 1 diarrhea. He received his 2nd AZD1222 vaccination on 09.06.21. On 15.06.21 the patient reported dyspnea on exertion, increased peripheral edema and general weakness. He was seen by our pulmonology service on 16.06.21 to rule out lung pathologies including pneumonia and radiation pneumonitis (patient had received radiation therapy in 2020).

Physical examination showed generalized edema, body weight increase of 2.77% (2kg). CT-examination showed bilateral pleural effusion and cardiomegaly. The patient then revealed that he had stopped taking all his cardiovascular medication 4-6 weeks prior including bisoprolol, amlodipin, ramipril, doxazosin, acetyl salicylic acid and atorvastatin. The patient did not disclose any reason for incompliance regarding his cardiological medication but retrospectively showed more fatigue during this period. The patient was put on diuretic therapy and scheduled for presentation at our cardiology department on the following day to gradually start his cardiac medication and monitor diuretic therapy given his aortic stenosis. However, the patient died before presenting in our cardiology department due to worsening of his cardiopulmonary function most likely caused by left ventricular dysfunction resulting from his aortic stenosis.

References:

1. J. Haanen *et al.*, Management of toxicities from immunotherapy: ESMO Clinical Practice Guidelines for diagnosis, treatment and follow-up. *Annals of oncology : official journal of the European Society for Medical Oncology* **28**, iv119-iv142 (2017).

Studienprotokoll Version 1.2 (02.07.2020)

Titel der Studie: **Analyzing the Therapeutically Induced Composition of Immunological Patterns Amid Tumor Eradication**

Studienleiter: Thomas Walle
Arzt in der Weiterbildung für Innere Medizin, Hämatologie und Onkologie
Nationales Centrum für Tumorerkrankungen (NCT) Heidelberg
Universitätsklinikum Heidelberg
Im Neuenheimer Feld 460
D-69120 Heidelberg
06221/56 38246
thomas.walle@med.uni-heidelberg.de

Weitere an der Studie Beteiligte:
Stellvertretender Studienleiter
Prof. Dr. med. Dr. rer. nat. Guy Ungerechts
Leitender Oberarzt und stellvertretender ärztlicher Direktor Medizinische Onkologie
Nationales Centrum für Tumorerkrankungen (NCT)
Im Neuenheimer Feld 460
D-69120 Heidelberg
06221/56-38718
guy.ungerechts@nct-heidelberg.de

Ambulant und stationär tätige Ärztinnen und Ärzte in den Ambulanzen und Tageskliniken des Universitätsklinikums Heidelberg.

Biometrische Beratung: Dr. sc. hum. Johannes Krisam
Institut für Medizinische Biometrie und Informatik
Im Neuenheimer Feld 130.3
69120 Heidelberg

Unterstützende Institutionen: Deutsches Krebsforschungszentrum
Im Neuenheimer Feld 280
69120 Heidelberg

Heidelberg, 20.06.2020 Thomas Walle

Summary

Immune checkpoint therapies have improved patient survival in many advanced solid tumors, but response rates are low in most indications. Retrospective studies suggest that similar peripheral blood immune cell subtypes expand in responders to different immunotherapies across several tumor types. Here, we seek to explore changes in the peripheral blood immune cell composition as histology agnostic on-treatment biomarker candidates across mono- and combination immunotherapies (IT) in a single-arm cohort trial with a retrospective training and prospective validation cohort. Cancer patients undergoing immunotherapy receive pre- and on-treatment peripheral blood draws (1-7 weeks) within the framework of the NCT Liquid Biobank. We will analyze changes in the immune contexture in an unbiased manner by single cell RNA sequencing, flow cytometry and quantitative serum proteomics. Based on these markers, we will develop a biomarker-guided multiparametric prediction model for radiological response to therapy, which will be the primary outcome of this study. Secondary outcomes include the immune cell and serum proteome/metabolome composition under IT and at disease progression, grade ≥3 adverse events, as well as progression-free and overall survival. This study will define tumor type independent on treatment-biomarkers, which can be validated in future prospective multicenter trials to guide cancer immunotherapy.

Contents

Summary 2

Introduction 4

Aim of the study 5

Outcome measures 5

Study Design 6

Time frame 8

Data protection 8

Risks for patients 8

Benefits for patients 9

Study type 9

Inclusion criteria 10

Exclusion criteria 10

Randomization 11

Exit criteria patients 11

Exit criteria study 11

Insurance for patients 11

Compensation for health damage as a result of the study interventions 11

Secure laboratory 11

Sample size estimation 11

Ethical aspects 13

Conflicts of interest 13

Funding 13

References 14

Attachments 15

Appendix 1 – Patient Information/Informed Consent Form of the NCT Liquid Biobank 17

Appendix 2 – Sample size estimation 28

Introduction

Immune-checkpoint inhibitors have prolonged overall survival in many types of tumor either in monotherapy or in combination with other immunotherapies, targeted therapies and/or cytotoxic chemo- or radiotherapy (1-6). However, only a small fraction of patients responds which generally cannot be identified reliably by available biomarkers such as immunohistochemistry assessment of PD-L1 and mismatch repair protein expression. Therefore, patients currently must undergo radiological imaging before and after 2-6 months of therapy to determine whether a relevant response has been achieved. In case of treatment failure, this results in a loss of time, which could be used for other promising therapies in these patients. Moreover, patients may suffer from unnecessary adverse events while undergoing an ineffective therapy for months.

Small retrospective studies suggest similar changes in peripheral blood mononuclear cells in responders to different immunotherapies in several types of tumor (7-10). A possible explanation for this observation is that different immunotherapies induce similar anti-tumor immune responses in multiple tumor types. These responses are not present before the start of therapy but can be exclusively detected after initiation of treatment. Therefore, changes in leukocyte composition during therapy represent a promising histology-agnostic on-treatment biomarker candidate across different immunotherapies. Because leukocyte phenotypes are shaped by the surrounding metabolomic and cytokine environment (11, 12), these factors should be accounted for when analyzing changes in immune cell composition.

Here, we will explore whether changes in peripheral blood immune cell composition and the serum proteome and metabolome can be used as early-on treatment biomarkers which predict response to immunotherapies across tumor types. These biomarkers could be used to identify non-responders to immunotherapies early and switch treatments in case of low response probability thereby saving patients valuable time and decreasing overall healthcare costs for expensive but in some cases ineffective therapies.

Another problem in most immunotherapies is the development of potentially life-threatening immune related adverse events (irAE) in which the immune response gets directed against healthy tissues. Currently, there are no biomarkers available predicting this event. As a secondary outcome we will therefore assess the peripheral blood immune cell composition and the surrounding serum proteome/metabolome as a possible biomarker for irAEs.

At some point of therapy patients will develop secondary resistance, meaning the progression of disease after an initial response. This may be associated with the development of immune escape mechanisms such as the recruitment of T cell suppressive cell populations or soluble mediators (13). We will characterize these changes at secondary resistance and thereby help identify new immunotherapy targets aiming to improve current immunotherapy approaches.

Aim of the study

Here we will analyze the immune cell, serum protein and metabolite composition in peripheral blood during immunotherapy (IT) and combinations of IT with other cancer therapies such as chemotherapy, radiotherapy, and targeted therapy across different types of tumor. We will derive a multiparametric predictive biomarker model for radiological response to IT based on changes in the abundances of different immune cell subsets, proteome and metabolite patterns during therapy. Moreover, we will develop predictive models for secondary therapy resistance and immune-related adverse events based on these biomarkers. To understand the molecular mechanisms leading to these events we will test different functions of peripheral blood immune cells of our patients using preclinical in vitro and xenograft mouse models.

Outcome measures

Primary outcome parameter

The study is exploratory in nature. The primary endpoint is radiological response to therapy for which a biomarker-based prediction model will be developed based on the immune cell composition and serum proteome and metabolome of these patients.

Secondary outcome parameters

Secondary endpoints include the immune cell and serum proteome and metabolome composition under IT and at disease progression, grade ≥3 adverse events including infections, as well as progression-free and overall survival. Select leukocyte subsets will be tested for cytotoxicity, migration, proliferation, metabolic function and cytokine production and differentiation properties in vitro and in xenograft mouse models. The effects of patient serum factors on these properties will also be investigated.

Study Design

Interventions:
This is a non-interventional cohort trial.

Patient recruitment and informed consent
Eligible are patients with malignant neoplasms (ICD-10 2016, C00-C97) undergoing immunotherapy at Heidelberg University Hospital, Heidelberg, Germany who receive blood draws before and 1-7 weeks after initiation of immunotherapy as well as every 8-12 weeks under immunotherapy within the framework of the “NCT Liquid Biobank” program. Patients recruited retrospectively provide informed consent upon inclusion into the “NCT Liquid Biobank” program and explicitly agree to cellular and genomic analyses (please see appendix 1). Patients were informed about the risks of identification especially regarding genomic analyses (appendix 1). It is therefore ethically justified to not ask these patients for study-specific consent. We will recruit 220 patients for the training cohort (including 10% drop-out), which may be recruited from the NCT liquid biobank retrospectively. We will recruit another 220 patients prospectively for the validation cohort (including 10% drop-out). These patients will provide written informed study-specific consent to “NCT ANTICIPATE”, Patient numbers are defined by statistical sample size estimation as outlined below (Appendix 2).

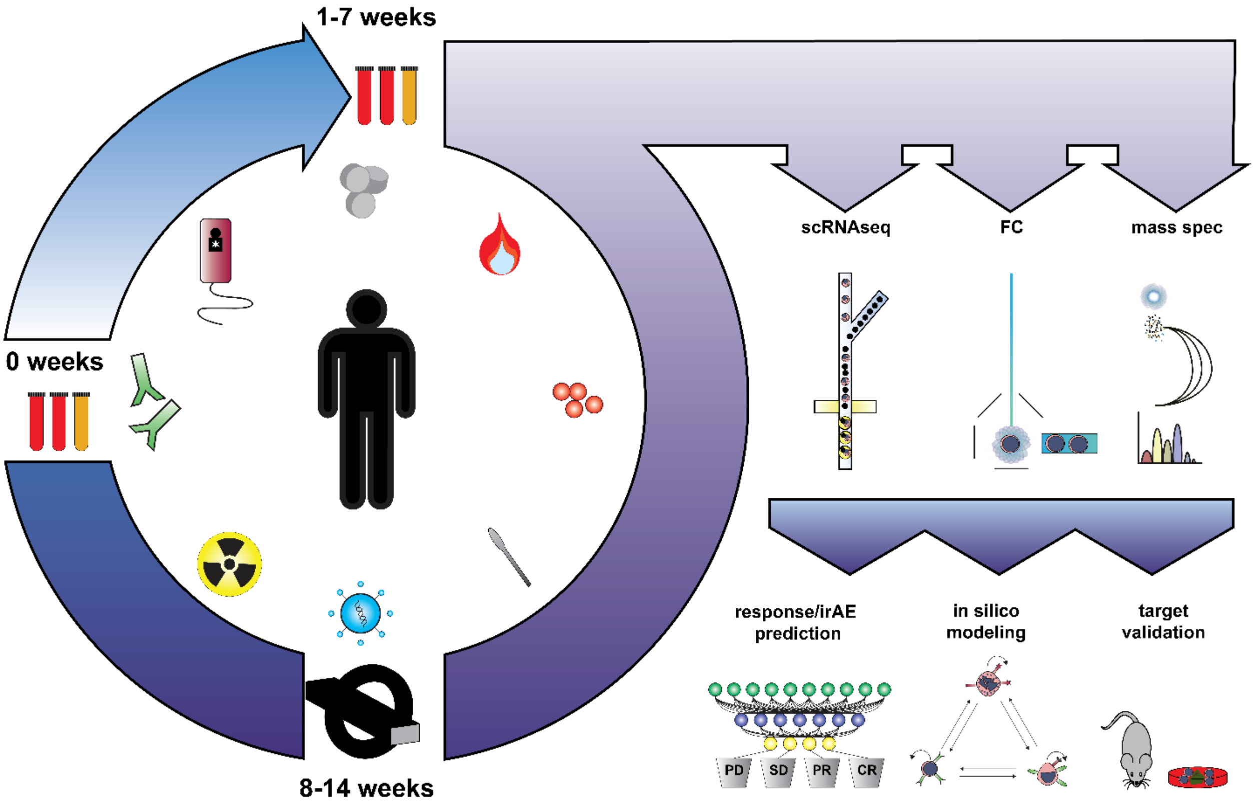

Figure 1 Sampling strategy

Patients of multiple tumor types undergoing immunotherapy will receive repeated blood draws at day 0, at 1-7 weeks and every 8-12 weeks after initiation of therapy until disease progression. Peripheral blood will be analyzed by single cell RNA sequencing (scRNAseq), flow cytometry and serum proteomics, as well as by in vitro and in vivo cytotoxicity, migration, metabolic function, proliferation and cytokine production and differentiation assays. These outcomes will be used to model radiological response to therapy as assessed by CT scan and development of immune related adverse events (irAEs).

Study outline

Peripheral blood mononuclear cells (PBMC) will be isolated by density gradient centrifugation at NCT Heidelberg laboratories. Samples will be stored in liquid N_2 . PBMC will be sorted for viable $CD45^+$ cells using fluorescence-assisted cell sorting and analyzed by single cell RNA sequencing and flow cytometry. Serum will be analyzed by quantitative mass spectrometry, multiplex cytokine and peptide arrays. Select protein concentrations changing under IT will be validated by ELISA and immunoblots. Radiological response to therapy will be determined using the RECIST 1.1 criteria (14). Changes in leukocyte subset frequencies, serum proteome and metabolome concentrations at 1-7 weeks after therapy initiation as

compared to baseline will be used to derive a predictive biomarker model for radiological response to therapy and a receiver-operator curve (ROC) will be calculated. This first model will be constructed using a training cohort of 200 patients which may be recruited retrospectively from the NCT liquid biobank. This model will be validated in a validation cohort of another 200 patients, which will be recruited prospectively.

To explore the biological relevance of specific leukocyte subsets PBMC will be challenged with different functional assays in vitro and after xenotransplantation in mice including measurements of cytotoxicity and its surrogates, proliferation, migration, metabolic function, cytokine production and differentiation. The effect of serum factors on these functions will also be investigated.

Time frame

This trial is planned for 7 years until 31.05.2027. We assume a recruitment of approximately 100 patients per year.

Data protection

Blood samples have been collected within the “NCT Liquid Biobank” in a pseudonymized format. The following information will be retrieved from Heidelberg University’s electronic patient record files: Whole blood counts and leukocyte differential counts as well as c-reactive protein at time of blood sample collection, age at day of first blood draw, gender (m/f/d), tumor type, stage and histology, mutational status of the tumor, sites of metastasis, ethnic background, history of tobacco use (current, previous, never), preexisting health conditions, concurrent medication and grade 3/4 adverse events during immunotherapies according to Common Terminology Criteria for Adverse Events (CTCAE) v.5.0 and the European Society for Medical Oncology guidelines on irAEs (15). Data retrieval will be performed by a physician or a Heidelberg University medical student who is in regular contact with oncology patients and is bound to „ärztliche Schweigepflicht“ and the Landesdatenschutzgesetz (LDSG BW, BDSG). The actual identity will only be known to the NCT biobank personal, the person retrieving the data and the study lead. All laboratory analyses will be performed under the patient pseudonym. Data collected within the trial include scRNAseq, FACS, protein and metabolite concentrations and leukocyte subset function, data from blood samples and imaging results. The timeframe for this study is set from 1.6.2020-31.05.2027. Data will be stored for 10 years after completion of the trial and will be anonymized on 01.06.2037. Patients have received contact numbers and e-mail addresses should they chose to resign from participation in the NCT liquid biobank program. In this case all information of the patient will be destroyed unless specified otherwise.

Risks for patients

This study is non-interventional. Risks are described in further detail in the “NCT Liquid Biobank” protocol (S-207/2005).

Benefits for patients

Participants do not directly benefit from participation in the trial. However, future patient generations may benefit from the results obtained in this study. Identification of a powerful on-treatment biomarker would enable faster identification of beneficial treatment strategies for individual patients and reduce the time of patients undergoing ineffective/suboptimal therapies with their inherent adverse events.

Study type

Single center non-interventional single-arm cohort study.

Inclusion criteria

- Fulfill inclusion criteria for "NCT Liquid Biobank"
- Patient age ≥ 18 years
- Retrospective patients: Written informed consent to "NCT Liquid Biobank" (Appendix 1)
- Prospective patients: Written informed consent to "NCT ANTICIPATE"
- Main diagnosis belongs to C00-C97 according to ICD-10 (2016), Advanced and/or inoperable and/or metastatic (M1 according to TNM 8th edition, AJCC)
- Measurable disease according to RECIST 1.1 (14)
- Undergoing an immunotherapy in monotherapy or in combination with other cancer-specific therapies. The following therapies are considered immunotherapies:
  - Immune checkpoint therapies are defined as a therapeutic compound targeting one of the following receptors or ligands: PD-1 (e.g. nivolumab, pembrolizumab); PD-L1 (e.g. avelumab, durvalumab, atezolizumab); PD-L2; CTLA-4 (e.g. ipilimumab, tremelimumab); GITR; GITRL; CD137; CD137L; OX40; OX40L; B7-H3; B7-H4; LIGHT; HVEM; CD70; CD27; CD28; CD80; CD86; CD40; CD40L; ICOS; LAG3; Galectin-3; CD155; TIGIT; CD226; CD96; VISTA; HVEM; BTLA; CD160; TIM-3; Galectin-9; CEACAM-1, IDO, CD47, SIRP-1α, CD73, CD39, TNFRSF and TNFSF members.
  - Cell based or acellular cancer vaccines
  - Virotherapies
  - Toll-like receptor modulators
  - Recombinant cytokines
  - T cell or Natural Killer (NK) cell engagers which are defined as therapeutic compounds bringing T cells or NK cells in proximity of tumor cells or tumor-associated stroma.
- complete sampling status (blood samples obtained before starting IT (baseline) and 1-7 weeks after therapy initiation) will be included in the final analysis. Patients may not have discontinued immunotherapy between baseline sample and 1-7 weeks sampling.
- Complete response evaluation: imaging studies by computed tomography or magnetic resonance imaging performed <6 weeks before start of the immune combination therapy (baseline) and 6-14 weeks after initiation. For inclusion in response analysis, patients may not have discontinued immunotherapy between the imaging studies used for response evaluation. Death from any cause will be considered progressive disease and may be used for response evaluation.

Exclusion criteria

- Passed exclusion criteria of „NCT Liquid Biobank"
- Adoptive effector cell therapies

- Including adoptive T/NK cell therapy, chimeric antigen receptor T cells or NK cells, tumor infiltrating leukocyte infusions)
- Not including adoptive dendritic cell transfers (dendritic cell based vaccines)

Randomization

This is a single-arm cohort trial. No randomization is required.

Exit criteria patients

- Withdrawal of consent for participation in the "NCT Liquid Biobank" or "NCT ANTICIPATE"
    - When patients decide to exit the study, they can decide whether their data and biomaterial collected up to this point can be used or whether all their personal data and biomaterials shall be destroyed.

Exit criteria study

- Publication of a tumor type and combination therapy independent biomarker predicting response to IT across histologies and above mentioned immunotherapies with an AUC of ≥0.90 within the 95% confidence interval in a prospective, independent, multicenter validation cohort.
- Failure to retrieve the required patient number for the training cohort by 1.6.2025

Insurance for patients

Not required

Compensation for health damage because of the study interventions

Following the "NCT Liquid Biobank" protocol no compensation is provided as no relevant health damage is inflicted.

Secure laboratory

All analyses will be performed and sample will be stored in rooms 02.116 and 99.101 at National Center for Tumor Diseases (NCT) Im Neuenheimer Feld 460 and rooms 01.116 and 01.113 at Otto-Meyerhof-Zentrum Im Neuenheimer Feld 350, 69120 Heidelberg.

Sample size estimation

Based on a meta-analysis of current clinically applied predictors to PD-1/PD-L1 immune checkpoint blockade (16), we defined an AUC of >0.78 as a clinically relevant threshold above the current biomarker predictive power. To assess the potential predictive power of using the immune cell composition as a biomarker we used published data from renal cell carcinoma patients undergoing combined nivolumab and ipilimumab immune checkpoint therapy to calculate a receiver operator curve **(Appendix 2, Figure 2)**. Based on this AUC confidence intervals for different numbers of patients were calculated assuming a response rate of 10% to immune checkpoint therapy. This response rate is the average response rate in poorly responding tumor types, which likely represent the majority of our patients at NCT (17). In our

sample size estimation, a patient number of n=200 resulted in a lower 95% confidence interval of AUC=0.782, which was above our predefined clinically relevant threshold. We estimate a drop-out of 10% due to loss to follow-up of the patients resulting in a total of 220 patients for the training and 220 patients for the validation cohorts to be recruited.

Statistical analysis

For the primary endpoint treatment response, a prediction model will be developed based on a logistic regression model with variable selection and compared to a deep neural network using a training cohort of 200 patients. Validity of the models will be assessed via cross-validation and further assessed in a prospective validation cohort of another 200 patients. The AUC for the prediction model will be calculated together with 95% confidence intervals. We will perform subset analyses for biomarker sensitivity and specificity in each tumor type and each combination therapy as well as after stratification for age, gender, tumor type, stage, histology, mutational status, sites of metastasis, ethnic background, preexistent health conditions, concurrent medication and ECOG. Secondary endpoints overall and progression-free survival will be evaluated using the Kaplan-Meier method. Differences in the distribution of the above-named variables between biomarker positive and negative patients between will be analyzed by Mann-Whitney-U tests (continuous, interval, ordinal variables) and chi-square tests (categorical variables).

Ethical aspects

The trial will be conducted in accordance with the declaration of Helsinki in its current edition. The study protocol will be evaluated by the ethics committee of the Medical Faculty of Heidelberg University. The names of the patients and other confidential or privileged information are in accordance with „ärztliche Schweigepflicht“, the Datenschutz-Grundverordnung (DSGVO) and the Landesdatenschutzgesetz (LDSG BW, BDSG). Confidential or privileged health related information will only be passed-on in a pseudonymized format. Third party members do not get insight into the original documentation unless required by law. Participation in the “NCT Liquid Biobank” is voluntary. Patients may withdraw consent by themselves or by their proxy by law at any time without reason and without disadvantages for their future treatment and healthcare. Patients were informed about the benefits and risks of participation in the “NCT Liquid Biobank” Participants were informed about the nature of the performed analysis on cellular compositions including genomic analyses. Patient consent has been documented and signed in the informed consent form in written form. This trial is supported financially by NCT Heidelberg. In case a patient withdraws consent of participation in the trial, all information obtained for this patient will be destroyed unless specified otherwise by the patient.

Conflicts of interest

All investigators have no conflicts of interest to disclose.

Funding

Cost will be covered by Clinical Cooperation Unit Virotherapy at National Center for Tumor Diseases (NCT) and German Cancer Research Center Heidelberg, Germany. Funding originates from public and private third parties.

References

1. Schmid P, Adams S, Rugo HS, Schneeweiss A, Barrios CH, Iwata H, et al. Atezolizumab and Nab-Paclitaxel in Advanced Triple-Negative Breast Cancer. New England Journal of Medicine. 2018;379(22):2108-21.
2. Formenti SC, Rudqvist NP, Golden E, Cooper B, Wennerberg E, Lhuillier C, et al. Radiotherapy induces responses of lung cancer to CTLA-4 blockade. Nature medicine. 2018.
3. Antonia SJ, Villegas A, Daniel D, Vicente D, Murakami S, Hui R, et al. Durvalumab after Chemoradiotherapy in Stage III Non-Small-Cell Lung Cancer. N Engl J Med. 2017.
4. Gandhi L, Rodriguez-Abreu D, Gadgeel S, Esteban E, Felip E, De Angelis F, et al. Pembrolizumab plus Chemotherapy in Metastatic Non-Small-Cell Lung Cancer. N Engl J Med. 2018.
5. Socinski MA, Jotte RM, Cappuzzo F, Orlandi F, Stroyakovskiy D, Nogami N, et al. Atezolizumab for First-Line Treatment of Metastatic Nonsquamous NSCLC. New England Journal of Medicine. 2018;378(24):2288-301.
6. Wolchok JD, Chiarion-Sileni V, Gonzalez R, Rutkowski P, Grob JJ, Cowey CL, et al. Overall Survival with Combined Nivolumab and Ipilimumab in Advanced Melanoma. N Engl J Med. 2017;377(14):1345-56.
7. Krieg C, Nowicka M, Guglietta S, Schindler S, Hartmann FJ, Weber LM, et al. High-dimensional single-cell analysis predicts response to anti-PD-1 immunotherapy. Nat Med. 2018.
8. Liakou CI, Kamat A, Tang DN, Chen H, Sun J, Troncoso P, et al. CTLA-4 blockade increases IFNgamma-producing CD4+ICOShi cells to shift the ratio of effector to regulatory T cells in cancer patients. Proc Natl Acad Sci U S A. 2008;105(39):14987-92.
9. Wei SC, Levine JH, Cogdill AP, Zhao Y, Anang NAS, Andrews MC, et al. Distinct Cellular Mechanisms Underlie Anti-CTLA-4 and Anti-PD-1 Checkpoint Blockade. Cell. 2017.
10. Goswami S, Walle T, Cornish AE, Basu S, Anandhan S, Fernandez I, et al. Immune profiling of human tumors identifies CD73 as a combinatorial target in glioblastoma. Nature medicine. 2019.
11. Teijeira A, Garasa S, Etxeberria I, Gato-Cañas M, Melero I, Delgoffe G. Metabolic Consequences of T-cell Costimulation in Anticancer Immunity. Cancer Immunology Research. 2019;7:1564-9.
12. Bunse L, Pusch S, Bunse T, Sahm F, Sanghvi K, Friedrich M, et al. Suppression of antitumor T cell immunity by the oncometabolite (R)-2-hydroxyglutarate2018.
13. Sharma P, Hu-Lieskovan S, Wargo JA, Ribas A. Primary, Adaptive, and Acquired Resistance to Cancer Immunotherapy. Cell. 2017;168(4):707-23.
14. Schwartz LH, Litiere S, de Vries E, Ford R, Gwyther S, Mandrekar S, et al. RECIST 1.1-Update and clarification: From the RECIST committee. Eur J Cancer. 2016;62:132-7.
15. Haanen J, Carbonnel F, Robert C, Kerr K, Peters S, Larkin J, et al. Management of toxicities from immunotherapy: ESMO Clinical Practice Guidelines for diagnosis, treatment and follow-up. Annals of oncology : official journal of the European Society for Medical Oncology. 2017;28:iv119-iv42.
16. Lu S, Stein JE, Rimm DL, Wang DW, Bell JM, Johnson DB, et al. Comparison of Biomarker Modalities for Predicting Response to PD-1/PD-L1 Checkpoint Blockade: A Systematic Review and Meta-analysis. JAMA Oncology. 2019;5(8):1195-204.
17. Yarchoan M, Hopkins A, Jaffee EM. Tumor Mutational Burden and Response Rate to PD-1 Inhibition. N Engl J Med. 2017;377(25):2500-1.

Attachments

1. Informed consent of the NCT Liquid Biobank
2. Informed consent of the NCT ANTICIPATE study
3. Statistical sample size estimation

Appendix 1 – Informed Consent Form of the NCT Liquid Biobank

Die NCT Biobank – eine humane Gewebe- und Flüssigbiobank am Nationalen Centrum für Tumorerkrankungen Heidelberg

Patienteninformation

Für den Verbleib beim Patienten

Sehr geehrte Patientin, sehr geehrter Patient,

Sie werden derzeit am Universitätsklinikum Heidelberg bzw. dem Nationalen Centrum für Tumorerkrankungen (NCT) behandelt. Unser Ziel ist es, Ihnen die bestmögliche Diagnostik und Therapie zukommen zu lassen. Wir sind ein national und international führender Standort in der Krebsbehandlung und der Krebsforschung. Menschliche Biomaterialien, wie Gewebe, Blut oder Urin sind eine wichtige Grundlage für diese Forschung. Sie werden zusammen mit ausgewählten medizinischen Daten in der Biobank des NCT aufbewahrt und für die Forschung zur Verfügung gestellt. Sie dienen dazu, die Vorbeugung, Erkennung und Behandlung von Tumorerkrankungen und deren Vorstufen zu verbessern.

Wir möchten Sie hiermit über die Ziele und die Verfahren der Biobank des NCT informieren, damit Sie sich eine eigene Meinung bilden und eine informierte Entscheidung treffen können. Anschließend bitten wir Sie, Ihre Proben und Daten der Biobank des NCT zur Verfügung zu stellen und damit die medizinische Forschung zu unterstützen.

Ihre Teilnahme ist freiwillig. Wenn Sie sich nicht beteiligen möchten, entstehen Ihnen keine Nachteile. Sie können Ihre Zustimmung jederzeit und ohne Angabe von Gründen widerrufen.

Wie werden die Bioproben gewonnen und um welche handelt es sich?

Gewebe: Im Rahmen Ihrer Behandlung werden Ihnen für die Diagnosestellung Gewebeproben entnommen. Jedes entnommene Gewebe wird mit höchster Sorgfalt und nach neuesten Erkenntnissen von einem Facharzt am Pathologischen Institut des Universitätsklinikums Heidelberg begutachtet. Danach würde nicht mehr benötigtes Gewebe (sog. „Restgewebe") sachgerecht entsorgt werden. Für die Forschung ist dieses Restgewebe jedoch von besonderer Bedeutung. Wir möchten daher um Ihre Zustimmung bitten, dieses Gewebe in der Biobank für Forschungszwecke sammeln und bearbeiten zu dürfen.

Blut: Sollte bei Ihnen im Rahmen der medizinischen Behandlung eine Blutentnahme vorgesehen sein, bitten wir Sie, maximal 30 ml Blut zusätzlich zu spenden. In Einzelfällen bitten wir Sie, für bestimmte zelluläre Analysen bis zu 110 ml Blut entnehmen zu dürfen. Die Entscheidung hierzu liegt nur bei Ihnen.

Andere Biomaterialien: Bei der Diagnostik und Behandlung ihrer Erkrankung können Ergussflüssigkeiten entnommen werden. Wir bitten Sie um Ihre Zustimmung, solche Materialien, die normalerweise entsorgt werden würden, in der Biobank lagern zu dürfen, um sie später für wissenschaftliche Untersuchungen verwenden zu können. Auch andere Biomaterialien (z.B. Abstriche, Spülflüssigkeiten, Speichel, Urin, Stuhl, Haare) können eine wichtige Grundlage für die medizinische Forschung darstellen, z.B. um den Verlauf einer Erkrankung zu untersuchen.

Alle Biomaterial-Spenden sind für Sie nicht mit zusätzlichen Untersuchungen (z.B. Röntgen-Aufnahmen) verbunden. Wir möchten Sie bitten, das Recht zur Nutzung an diesen Proben an die Biobank des NCT zu übertragen. Sie können die Zustimmung zur Nutzung der Proben jederzeit widerrufen.

Was passiert mit meinen Biomaterialien und Daten?

Die von Ihnen zur Verfügung gestellten Biomaterialien und Daten werden ausschließlich für die biomedizinische Forschung verwendet. Das Hauptziel ist es, ein besseres Verständnis der Entstehung und Ausbreitung von Krebserkrankungen zu erreichen sowie neue Diagnose- und Therapieansätze zu finden. Die Untersuchungen werden mit modernsten Labormethoden durchgeführt und können z.B. umfangreiche Analysen des Erbguts, der Gewebezusammensetzung oder der Steuerung von Zellen umfassen. Die Blut- oder Gewebeproben werden hierfür nach höchsten Qualitätsstandards so lange aufbewahrt, bis das Material verbraucht ist oder nicht mehr den Qualitätskriterien für eine wissenschaftliche Weiterverwendung genügt Die langfristige Aufbewahrung soll es ermöglichen, auch erst in Zukunft entwickelte Untersuchungsmethoden anwenden zu können. Zum derzeitigen Zeitpunkt können daher noch nicht alle zukünftigen medizinischen Forschungsziele beschrieben werden. Es kann also sein, dass Ihre Proben und Daten auch für Forschungsfragen verwendet werden, die wir heute noch nicht absehen können.

Im Rahmen von Forschungsaktivitäten arbeiten wir auch überregional mit anderen wissenschaftlichen Einrichtungen zusammen. Dies können Universitäten, Kliniken, außeruniversitäre Einrichtungen oder Forschungsnetzwerke sein, in fest definierten Forschungsprojekten auch industrielle Partner im In- und Ausland. Dies kann auch Länder betreffen, in denen die Datenschutzanforderungen niedriger sind als in der Europäischen Union. Die Studienleitung wird alle angemessenen Schritte unternehmen, um den Schutz Ihrer Proben und Daten gemäß den Datenschutzstandards der Europäischen Union zu gewährleisten. Ihre Biomaterialien und Daten geben wir nur innerhalb dieser Kooperationen und nach genauer Prüfung des Projektvorhabens an autorisierte Personen weiter. Sie dürfen nur für den vorbestimmten Forschungszweck verwendet und vom Empfänger nicht an Dritte weitergegeben werden. Hierbei nicht verbrauchtes Material wird an die Biobank zurückgegeben oder vernichtet. Um die Zusammenarbeit innerhalb dieser Kooperationen zu erleichtern, kann es mitunter erforderlich sein, ihre Daten an eine zentrale Stelle zu übermitteln, wo sie umgehend in einen verschlüsselten Code übersetzt werden. Eine sorgfältige Verschlüsselung gewährleistet, dass kein mit Ihren Proben und/oder Daten arbeitender Wissenschaftler über Ihre Identität informiert wird. Es ist alles Forschern ausdrücklich untersagt, irgendwelche Versuche zu unternehmen, um die Identität der Proben und/oder Daten in Erfahrung zu bringen.

Wenn Sie mit der beschriebenen Art und Dauer der Nutzung nicht in vollem Umfang einverstanden sind, werden Ihre Biomaterialien und Daten nicht für die Biobank verwendet.

Habe ich Nachteile, wenn ich an der Biobank teilnehme?

Eine Teilnahme ist für Sie mit keinen erkennbaren Nachteilen oder zusätzlichem Aufwand verbunden. Ihre derzeitige Diagnostik oder Behandlung als auch alle zukünftigen Behandlungen werden dadurch nicht beeinflusst. Es wird ausdrücklich darauf hingewiesen, dass Ihre Teilnahme freiwillig ist.

Habe ich Vorteile, wenn ich an der Biobank teilnehme?

Persönlich können Sie keinen Nutzen oder Vorteil aus der Spende Ihrer Proben und Daten erwarten. Die Proben und Daten sind ausschließlich für Forschungszwecke bestimmt. Eine Rückmeldung von Ergebnissen aus Untersuchungen der Biomaterialien ist daher nicht vorgesehen. Eine finanzielle Vergütung für entnommene Bioproben kann und darf nicht erfolgen. Sollte aus den Forschungserkenntnissen ein späterer kommerzieller Nutzen (z.B. im Rahmen einer neuen Medikamentenentwicklung) erzielt werden, sind weder Sie noch die Biobank daran beteiligt.

Welcher Nutzen ergibt sich für die Gesellschaft?

Alle derzeit durchgeführten wie auch zukünftigen medizinisch-wissenschaftlichen Forschungsvorhaben zielen auf eine Verbesserung unseres Verständnisses für die Entstehung, die Diagnose und damit die Neuentwicklung und Verbesserung von Behandlungsansätzen von Krebs und dessen Vorstufen sowie begünstigenden Erkrankungen. Mit Ihrem Einverständnis zur Übernahme Ihrer Biomaterialien in die Biobank des NCT leisten Sie einen wichtigen Beitrag zur Forschung und damit auch zur weiteren Verbesserung unseres Wissens, der Entwicklung neuer und besserer Diagnose- und Therapieverfahren und damit auch der medizinischen Versorgung. Informationen über die Aktivitäten der Biobank des NCT finden Sie unter **www.biobank-heidelberg.de**.

Welche Risiken sind mit Ihrer Spende verbunden?

Gesundheitliche Risiken: Die Gewebeentnahme oder Punktion von Ergüssen erfolgt im Rahmen einer ohnehin vorgesehenen diagnostischen oder therapeutischen Maßnahme (Biopsie oder einer Operation) und ist deshalb für Sie mit keinem zusätzlichen gesundheitlichen Risiko verbunden. Blutentnahmen erfolgen im Rahmen der erforderlichen diagnostischen Entnahmen, so dass Risiken und Belastungen durch zusätzlich entnommenes Blut als extrem gering einzustufen sind. Bei ausgeprägter Blutarmut (Hb <8 g/dl) wird auf die Entnahme von zusätzlichem Blut verzichtet.

Sonstige Risiken: Bei jeder Erhebung, Speicherung und Übermittlung von Daten besteht ein nicht gänzlich auszuschließendes Restrisiko, Sie zu identifizieren. Dies gilt insbesondere im Hinblick auf Ihre Erbsubstanz (genetische Daten). In der Biobank des NCT werden Ihre personen- und krankheitsbezogenen Daten deshalb getrennt voneinander und unter Berücksichtigung aller Datenschutzmaßnahmen gespeichert, um dieses Risiko zu minimieren. Dennoch lassen sich nicht alle Risiken restlos ausschließen, insbesondere wenn Sie selbst Ihre (genetischen) Daten im Internet veröffentlichen (z.B. zur Ahnenforschung).

Die Biobank des NCT versichert Ihnen jedoch, alles nach dem Stand der Technik Mögliche zum Schutz Ihrer Privatsphäre zu unternehmen. Proben und Daten werden nur an zur strikten Vertraulichkeit verpflichtete und befugte Personen und nur für klar definierte und schriftlich zu beantragende Projektvorhaben weiter gegeben.

Wie werden meine Biomaterialien und Daten geschützt?

Die Biobank des NCT arbeitet nach strengen Regeln des Datenschutzes, das heißt alle mit der Datenprüfung beauftragten Personen sind zur strengen Vertraulichkeit und zur Beachtung des Datenschutzes verpflichtet. Voraussetzung für die Gewinnung und Nutzung Ihrer Körpermaterialien ist zunächst einmal Ihre Einwilligung, welche freiwillig ist und jederzeit widerrufen werden kann. Alle unmittelbar Ihre Person identifizierenden Daten (Name, Geburtsdatum, Anschrift etc.) werden unverzüglich pseudonymisiert, das heißt durch eine zufällige Kombination aus Zahlen und Buchstaben ersetzt, die keinen Bezug zu Ihrer Person haben. Ihre Biomaterialien und medizinischen Daten werden dann getrennt von den identifizierenden Daten in der Biobank des NCT unter hohen Qualitäts- und Sicherheitsstandards aufbewahrt. Eine Verbindung zwischen Ihrem Namen und den medizinischen Daten ist somit nur über eine Entschlüsselung durch autorisierte Personen in zwei unterschiedlichen Systemen möglich. Eine Weitergabe von Daten an unberechtigte Dritte, etwa Versicherungsunternehmen oder Arbeitgeber, erfolgt nicht.

Wissenschaftliche Veröffentlichungen von Ergebnissen erfolgen ausschließlich anonymisiert, also in einer Form, die keine Rückschlüsse auf Ihre Person zulässt.

Sie haben das Recht, Auskunft über die von Ihnen gespeicherten personenbezogenen Daten zu verlangen. Ebenfalls können Sie die Berichtigung unzutreffender Daten sowie die Löschung der Daten oder Einschränkung deren Verarbeitung verlangen.

Der Verantwortliche für die Erhebung personenbezogener Daten für die NCT Biobank ist:

Nationales Centrum für Tumorerkrankungen (NCT)
Universitätsklinikum Heidelberg
Im Neuenheimer Feld 460
69120 Heidelberg

Tel.: 06221-56-7228
Fax: 06221-56-7225

Bei Anliegen zur Datenverarbeitung und zur Einhaltung der datenschutzrechtlichen Anforderungen können Sie sich an folgende Adresse wenden:

Datenschutzbeauftragter der Universitätsklinikums Heidelberg:
Im Neuenheimer Feld 672
69120 Heidelberg
Tel.:06221-56-7036

E-Mail: datenschutz@med.uni-heidelberg.de

Im Falle einer rechtswidrigen Datenverarbeitung haben Sie das Recht, sich bei folgender Aufsichtsbehörde zu beschweren:

Der Landesbeauftragte für den Datenschutz und die Informationsfreiheit Baden-Württemberg
Postfach 10 29 32, 70025 Stuttgart
Königstraße 10a, 70173 Stuttgart
Tel.: 0711-61 55 41-0
Fax: 0711-61 55 41-15
E-Mail: poststelle@lfdi.bwl.de
Internet: http://www.baden-wuerttemberg.datenschutz.dea.

Was beinhaltet Ihr Widerrufsrecht?

Sie können Ihre Einwilligung zur Verwendung Ihrer Biomaterialien und Daten jederzeit und ohne Angabe von Gründen widerrufen. In diesem Fall werden nach Wunsch entweder nur ihre zukünftig entnommenen Körpermaterialien nicht weiter gelagert und verwendet oder es können auch alle bereits gesammelten Körpermaterialien vernichtet und zugehörige Daten gelöscht werden. Über die Vernichtung der Proben bzw. Löschung der Daten werden Sie informiert. Daten bereits durchgeführter Analysen können zwar nicht mehr gelöscht, aber auch nicht mehr Ihrer Person zugeordnet werden. Bei einem Widerruf wenden Sie sich unter Angabe Ihrer Daten (Vorname, Nachname, Geburtsdatum) an:

Biobank des NCT
Institut für Pathologie
Universitätsklinikum Heidelberg
Im Neuenheimer Feld 224
69120 Heidelberg
Info.bmbh@med.uni-heidelberg.de

Was mache ich, wenn ich noch weitere Fragen habe?

Sollte Ihnen noch etwas unklar sein, wenden Sie sich bitte an die Sie aufklärende Person oder Ihre/n behandelnde/n Arzt/Ärztin. Bei Fragen bezüglich der Biobank können Sie das Sekretariat der NCT Biobank (Telefon Nr.: 06221-56-35215) kontaktieren. Ihre Fragen werden umgehend an einen sachkundigen Mitarbeiter weitergegeben. Weitere Informationen finden Sie zusätzlich unter **www.biobank-heidelberg.de.**

Indem Sie beiliegende Einverständniserklärung unterschreiben, geben Sie Ihre Zustimmung zur oben beschriebenen Handhabung Ihrer Proben und Daten.

Wir danken Ihnen für Ihre Unterstützung und wünschen Ihnen eine gute Genesung!

Leerseite für doppelseitigen Druck

Einwilligungserklärung

Zur Spende, Lagerung und Nutzung von Bioproben und klinischen Daten für die medizinische Forschung durch die Biobank des NCT

Patient/in (Name, Vorname): ....

geboren am ....

Für den Verbleib beim Patienten

Hiermit erkläre ich, dass ich verständlich über die Biobank des NCT, die Verwendung meiner Proben und Daten sowie die damit verbundenen Risiken aufgeklärt worden bin. Ich habe den Text der Patienteninformation erhalten und sowohl diesen als auch die Einverständniserklärung gelesen und verstanden. Aufgetretene Fragen wurden mir verständlich und ausreichend beantwortet.

Ich bin damit einverstanden, dass meine Bioproben (Gewebe, Blut, Urin oder anderes), wie in der Informationsschrift beschrieben, entnommen, sachgerecht gelagert und bis zum vollständigen Verbrauch des Materials, dessen Vernichtung aufgrund von Qualitätseinschränkungen oder meinem Widerruf für medizinische Forschungsvorhaben verwendet werden. Das Nutzungsrecht an diesen Biomaterialien übertrage ich der Biobank des NCT.

Meine Teilnahme ist **freiwillig** und ich habe jederzeit das Recht, die Einwilligung zur Sammlung und Nutzung meiner Biomaterialien ohne Angaben von Gründen zu widerrufen. Mir entstehen keine Nachteile durch den Widerruf. Meine Proben werden daraufhin vernichtet und die zugehörenden Daten werden gelöscht; Daten, die schon für Forschungsprojekte verwendet wurden, können nicht gelöscht werden.

Um eine korrekte Dokumentation der Daten zu gewährleisten, dürfen zur Verschwiegenheit verpflichtete Personen Einblick in meine personenbezogenen Krankheitsdaten nehmen, soweit diese vorliegen und sofern dies erforderlich ist. Für diesen Zweck entbinde ich die behandelnden Ärzte von der ärztlichen Schweigepflicht.

Ich erhalte keine Information über Forschungsergebnisse oder Zufallsbefunde. Zudem wurde ich darüber informiert, dass die Teilnahme an der Biobank des NCT für mich keinen persönlichen Vorteil oder kommerziellen Nutzen beinhaltet. Alle Rechte, die mit der Entwicklung neuer Therapien und Diagnostika, neuen Erkenntnissen oder der Entstehung schützenswerten Eigentums verbunden sind, übertrage ich der Biobank des NCT und den beteiligten Forschungspartnern.

Datenschutzerklärung:

Ich erkläre mich damit einverstanden, dass die Biobank des NCT, wie beschrieben,

- **Personenbezogene Daten von mir erhebt und speichert,**
- **Weitere Angaben aus meinen Krankenunterlagen entnimmt,**
- **Die Daten gemeinsam mit meinen Biomaterialien nur in verschlüsselter Weise für medizinische Forschungsvorhaben zur Verfügung stellt.**

Ich habe das Recht, Auskunft (einschließlich unentgeltlicher Überlassung einer Kopie) über die mich betreffenden personenbezogenen Daten zu erhalten sowie ggf. deren Berichtigung oder Löschung zu verlangen.

Ich möchte die Verwendung meiner Daten für andere/künftige Forschungszwecke wie folgt eingrenzen:

____

____

Ich stimme den oben genannten Punkten zu. Eine Kopie der Patienten-/Probandeninformation und Einwilligungserklärung habe ich erhalten. Das Original verbleibt bei der Biobank des NCT.

_______________ ____

Ort, Datum (vom Patienten/Probanden einzutragen), Unterschrift des Patienten

Ich habe das Aufklärungsgespräch geführt und die Einwilligung des Patienten eingeholt.

_______________ ____

Name des Arztes Name des Arztes/der aufklärenden Person in Druckbuchstaben

_______________ ____

Ort, Datum, Unterschrift des Arztes/ der aufklärenden Person

Patientenaufkleber

Einwilligungserklärung

Zur Spende, Lagerung und Nutzung von Bioproben und klinischen Daten für die medizinische Forschung durch die Biobank des NCT

Patient/in (Name, Vorname): ....,

geboren am ....

Hiermit erkläre ich, dass ich verständlich über die Biobank des NCT, die Verwendung meiner Proben und Daten sowie die damit verbundenen Risiken aufgeklärt worden bin. Ich habe den Text der Patienteninformation erhalten und sowohl diesen als auch die Einverständniserklärung gelesen und verstanden. Aufgetretene Fragen wurden mir verständlich und ausreichend beantwortet.

Ich bin damit einverstanden, dass meine Bioproben (Gewebe, Blut, Urin oder anderes), wie in der Informationsschrift beschrieben, entnommen, sachgerecht gelagert und bis zum vollständigen Verbrauch des Materials, dessen Vernichtung aufgrund von Qualitätseinschränkungen oder meinem Widerruf für medizinische Forschungsvorhaben verwendet werden. Das Nutzungsrecht an diesen Biomaterialien übertrage ich der Biobank des NCT.

Meine Teilnahme ist **freiwillig** und ich habe jederzeit das Recht, die Einwilligung zur Sammlung und/oder der Verarbeitung und Nutzung meiner Biomaterialien ohne Angaben von Gründen zu widerrufen. Mir entstehen keine Nachteile durch den Widerruf. Meine Proben werden daraufhin vernichtet und meine Daten werden gelöscht; Daten, die schon für Forschungsprojekte verwendet wurden, können nicht gelöscht werden.

Um eine korrekte Dokumentation der Daten zu gewährleisten, dürfen zur Verschwiegenheit verpflichtete Personen Einblick in meine personenbezogenen Krankheitsdaten nehmen, soweit diese in der Klinik vorliegen und sofern dies erforderlich ist. Für diesen Zweck entbinde in die behandelnden Ärzte von der ärztlichen Schweigepflicht.

Ich erhalte keine Information über Forschungsergebnisse oder Zufallsbefunde. Zudem wurde ich darüber informiert, dass die Teilnahme an der Biobank des NCT für mich keinen persönlichen Vorteil oder kommerziellen Nutzen beinhaltet. Alle Rechte, die mit der Entwicklung neuer Therapien und Diagnostika, neuen Erkenntnissen oder der Entstehung schützenswerten Eigentums verbunden sind, übertrage ich der Biobank des NCT und den beteiligten Forschungspartnern.

Für den Verbleib in der Klinik

08_Sonstige
108099

Datenschutzerklärung:

Ich erkläre mich damit einverstanden, dass die Biobank des NCT, wie beschrieben,

- **Personenbezogene Daten von mir erhebt und speichert,**
- **Weitere Angaben aus meinen Krankenunterlagen entnimmt,**
- **Die Daten gemeinsam mit meinen Biomaterialien nur in verschlüsselter Weise für medizinische Forschungsvorhaben zur Verfügung stellt.**

Ich habe das Recht, Auskunft (einschließlich unentgeltlicher Überlassung einer Kopie) über die mich betreffenden personenbezogenen Daten zu erhalten sowie ggf. deren Berichtigung oder Löschung zu verlangen.

Ich möchte die Verwendung meiner Daten für andere/künftige Forschungszwecke wie folgt eingrenzen:

____

____

Ich stimme den oben genannten Punkten zu. Eine Kopie der Patienten-/Probandeninformation und Einwilligungserklärung habe ich erhalten. Das Original verbleibt bei der Biobank des NCT.

Für den Verbleib in der Klinik

Ich stimme den oben genannten Punkten zu. Eine Kopie der Patienten-/Probandeninformation und Einwilligungserklärung habe ich erhalten. Das Original verbleibt bei der Biobank des NCT.

_______________ ____

Ort, Datum (vom Patienten/Probanden einzutragen), Unterschrift des Patienten

Ich habe das Aufklärungsgespräch geführt und die Einwilligung des Patienten eingeholt.

_______________ ____

Name des Arztes/der aufklärenden Person in Druckbuchstaben

_______________ ____

Ort, Datum, Unterschrift des Arztes/der aufklärenden Person

08_Sonstige
1080099

Appendix 2:

See attached

Appendix 3 – Sample size estimation

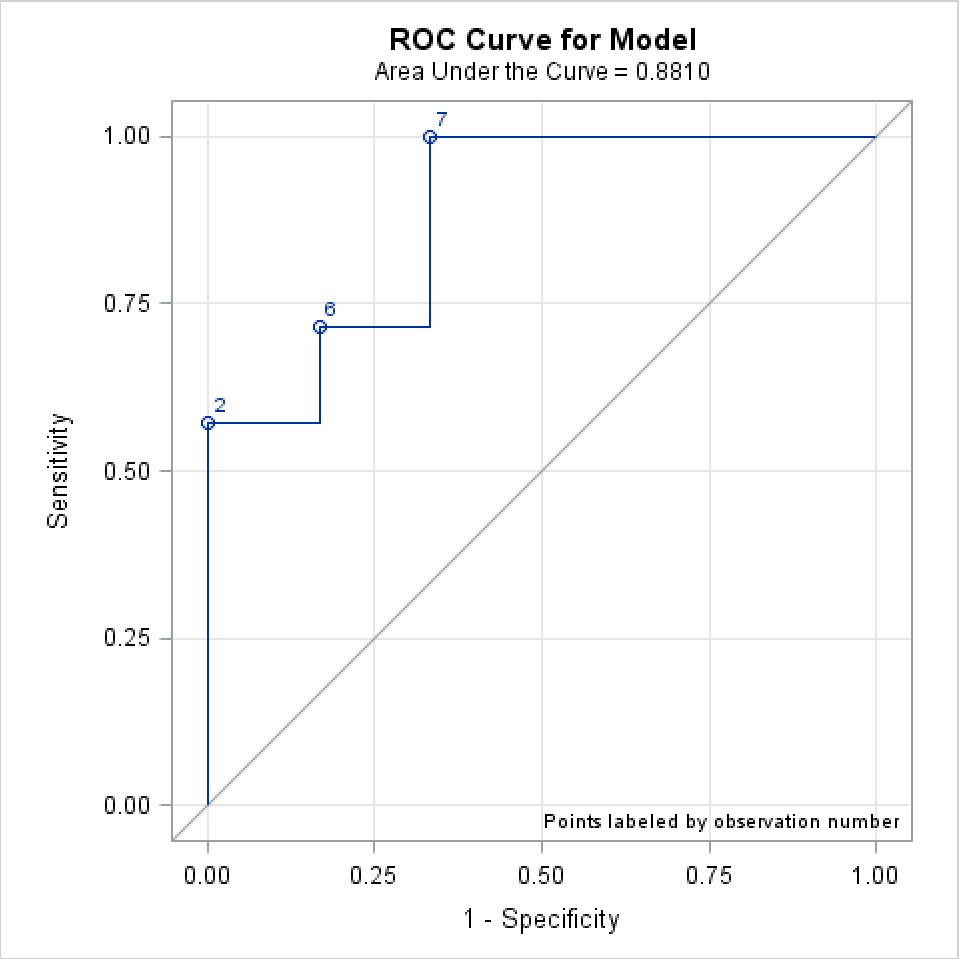

Figure 2 Receiver-operator curve of the dataset used for sample size estimation

Receiver-operator curve calculated on fold changes of an immune cell subset in responders and non-responders to combined ipilimumab and nivolumab therapy in an published exemplary dataset. (Extended Data Figure 3B, Cluster P24) (10). Calculations were performed using PASS 16.0.3 NCSS Statistical Software.

Confidence Intervals for the Area Under an ROC Curve

Numeric Results for Two-Sided Confidence Interval for ROC Curve's AUC

Confidence Level	Total Subjects N	Ratio N2/N1 R	Number Positive N1	Number Negative N2	Sample AUC	C.I. Width UCL-LCL	Lower Conf Limit LCL	Upper Conf Limit UCL
0,950	200	9,000	20	180	0,881	0,197	0,782	0,980

Report Definitions

Confidence Level is the proportion of confidence intervals (constructed with this same confidence level, sample size, etc.) that would contain the true coefficient alpha.
N is the total number of subjects sampled.
R is N2 / N1, so that N2 = R x N1.
N1 is the number of subjects sampled from the 'positive' group.
N2 is the number of subjects sampled from the 'negative' group.
Sample AUC is the anticipated value of the sample area under the ROC curve.
C.I. Width (UCL-LCL) is the width of the confidence interval. It is the distance from the lower limit to the upper limit.
Lower and Upper Confidence Limits are the actual limits that would result from a dataset with these statistics. They may not be exactly equal to the specified values because of the discrete nature of the N1 and N2.

References

Hanley, J.A. and NcNeil, B.J. 1982. 'The Meaning and Use of the Area under a Receiver Operating Characteristic (ROC) Curve.' Radiology, Vol 148, 29-36.
Kryzanowski, W.J. and Hand, D.J. 2009. 'ROC Curves for Continuous Data.' Chapman & Hall/CRC Press.

Calculations were performed using PASS 16.0.3 NCSS Statistical Software.
