# Supplementary material for: Cytokine release syndrome-like serum responses after COVID-19 vaccination are frequent and clinically inapparent under cancer immunotherapy
Source: Nat Cancer. 2022 Jun 17;3(9):1039–51. doi: 10.1038/s43018-022-00398-7 (PMC9499865; doi:10.1038/s43018-022-00398-7)
Supplement: Supplementary file 2 — Reporting Summary [file 43018_2022_398_MOESM2_ESM.pdf]

nature portfolio

Corresponding author(s): Thomas Walle

Last updated by author(s): May 12, 2022

Reporting Summary

Nature Portfolio wishes to improve the reproducibility of the work that we publish. This form provides structure for consistency and transparency in reporting. For further information on Nature Portfolio policies, see our Editorial Policies and the Editorial Policy Checklist.

Statistics

For all statistical analyses, confirm that the following items are present in the figure legend, table legend, main text, or Methods section.

n/a	Confirmed	
☐	☒	The exact sample size (*n*) for each experimental group/condition, given as a discrete number and unit of measurement
☐	☒	A statement on whether measurements were taken from distinct samples or whether the same sample was measured repeatedly
☐	☒	The statistical test(s) used AND whether they are one- or two-sided<br>*Only common tests should be described solely by name; describe more complex techniques in the Methods section.*
☐	☒	A description of all covariates tested
☐	☒	A description of any assumptions or corrections, such as tests of normality and adjustment for multiple comparisons
☐	☒	A full description of the statistical parameters including central tendency (e.g. means) or other basic estimates (e.g. regression coefficient) AND variation (e.g. standard deviation) or associated estimates of uncertainty (e.g. confidence intervals)
☐	☒	For null hypothesis testing, the test statistic (e.g. *F*, *t*, *r*) with confidence intervals, effect sizes, degrees of freedom and *P* value noted<br>*Give P values as exact values whenever suitable.*
☐	☒	For Bayesian analysis, information on the choice of priors and Markov chain Monte Carlo settings
☒	☐	For hierarchical and complex designs, identification of the appropriate level for tests and full reporting of outcomes
☐	☒	Estimates of effect sizes (e.g. Cohen's *d*, Pearson's *r*), indicating how they were calculated

Our web collection on statistics for biologists contains articles on many of the points above.

Software and code

Policy information about availability of computer code

Data collection

Data was collected from patient electronic health records or FCS files generated using BD FACS DIVA v8.0.

Data analysis

All custom code is provided under an MIT license https://doi.org/10.5281/zenodo.6544522
Data was analyzed in python 3 using Jupyter Notebook (v1.0.0) and included the following open source software packages:
imgaug==0.2.5
matplotlib==3.4.3
pandas==0.23.4
squarify==0.4.3
lifelines==0.26.4
statsmodels==0.10.2
numpy==1.19.5
seaborn==0.11.2
mpmath==1.2.1
scipy==1.4.1
pymatch==0.3.4
jupyter==1.0.0

Flow cytometry data was analyzed using Legendplex Software v 2021.07.01 (Biolegend, San Diego, CA, U.S.A.).
Statistical analysis and plotting was performed with GraphPad Prism 9.2.0 (GraphPad Software, San Diego, CA, U.S.A.).
Multiple comparison p value correction was performed with R 4.1.1
Plots were arranged using Adobe Illustrator 2021 (25.2.2, Adobe Inc, San Jose, CA, USA).

All custom computer code used to generate the results in this study has been deposited in a GitHub repository (https://github.com/wallet-maker/ANTICIPATE_COVID-19.git) and a Zenodo repository (https://doi.org/10.5281/zenodo.6544522) under an MIT license.

For manuscripts utilizing custom algorithms or software that are central to the research but not yet described in published literature, software must be made available to editors and reviewers. We strongly encourage code deposition in a community repository (e.g. GitHub). See the Nature Portfolio guidelines for submitting code & software for further information.

Data

Policy information about availability of data

All manuscripts must include a data availability statement. This statement should provide the following information, where applicable:

- Accession codes, unique identifiers, or web links for publicly available datasets
- A description of any restrictions on data availability
- For clinical datasets or third party data, please ensure that the statement adheres to our policy

The patient data that support the findings of this study are available in in pseudonymized form open access in zenodo with the identifiers: 10.5281/zenodo.6544522 . This includes clinical metadata including survival as well as primary data on cytokine concentrations and neutralizing antibody concentrations. Data is available until 8th December 2031 for 10 years as consented to by the study participants. The trial has been prospectively registered and the study protocol including the statistical analysis plan is available under the German Clinical Trial Register (https://drks.de) under the accession number DRKS00022890. No custom materials have been generated during this study.

Field-specific reporting

Please select the one below that is the best fit for your research. If you are not sure, read the appropriate sections before making your selection.

☒ Life sciences ☐ Behavioural & social sciences ☐ Ecological, evolutionary & environmental sciences

For a reference copy of the document with all sections, see nature.com/documents/nr-reporting-summary-flat.pdf

Life sciences study design

All studies must disclose on these points even when the disclosure is negative.

Sample size

Sample size estimation was performed for the primary outcome of this study prediction of radiological response which will be reported elsewhere. Based on a meta-analysis of current clinically applied predictors to PD-1/PD-L1 immune checkpoint blockade (10.1001/jamaoncol.2019.1549), we defined an AUC of >0.78 as a clinically relevant threshold above the current biomarker predictive power. To assess the potential predictive power of using the immune cell composition as a biomarker we used published data from renal cell carcinoma patients undergoing combined nivolumab and ipilimumab immune checkpoint therapy to calculate a receiver operator curve (10.1038/s41591-019-0694-x). Based on this AUC confidence intervals for different numbers of patients were calculated assuming a response rate of 10% to immune checkpoint therapy. This response rate is the average response rate in poorly responding tumor types, which likely represent the majority of our patients at our center. In our sample size estimation, a patient number of n=200 resulted in a lower 95% confidence interval of AUC=0.782, which was above our predefined clinically relevant threshold. We estimate a drop-out of 10% due to loss to follow-up of the patients resulting in a total of 220 patients for the training/exploration cohort reported here.

Data exclusions

We excluded one patient who had an immune related adverse event (arthritis grade 3) at baseline before vaccination because this not vaccine related event could have confounded immunological analyses

Replication

Cytokine concentrations and neutralizing antibody concentrations were measured in duplicates. Cytokine concentrations were measured in 3 independent experiments on different patients all with similar results (replication successful). Antibody concentrations were assessed in one experiments.

Randomization

This is a cohort study, no randomization was performed. We used a multivariate Cox proportional hazards model to assess the effects of different covariates (ECOG, line of therapy, age, mono vs combination immunotherapy, sex, tumor type, tumor stage, insurance status, vaccination status) on survival outcomes. For adjusting covariates for adverse event analysis we performed propensity score matching with the propensity score calculated either on sex, age and ECOG or on sex, age and insurance status.

Blinding

For cytokine concentration data the acquisition and processing of the raw data was performed by a clinician scientist who was blinded to the patients' identity and metadata and who was not involved in downstream data analysis. Notably, the patients' pseudonyms contained the temporal sequence of the samples.

Reporting for specific materials, systems and methods

We require information from authors about some types of materials, experimental systems and methods used in many studies. Here, indicate whether each material, system or method listed is relevant to your study. If you are not sure if a list item applies to your research, read the appropriate section before selecting a response.

Materials & experimental systems

n/a	Involved in the study
☐	☒ Antibodies
☒	☐ Eukaryotic cell lines
☒	☐ Palaeontology and archaeology
☒	☐ Animals and other organisms
☐	☒ Human research participants
☐	☒ Clinical data
☒	☐ Dual use research of concern

Methods

n/a	Involved in the study
☒	☐ ChIP-seq
☐	☒ Flow cytometry
☒	☐ MRI-based neuroimaging

Antibodies

Antibodies used

Legendplex Cytokine Storm Panel 1 (AB_2895549 (antibodyregistry.org), 741091, Biolegend, CA, USA), Cytokine Storm Panel 2 (AB_2895550 (antibodyregistry.org), 741142, Biolegend, CA, USA), SARS-CoV-2 Neutralizing Antibody Assay (AB_2895551 (antibodyregistry.org), 741127, Biolegend, CA, USA)

Validation

The antibodies of the Cytokine Storm Panels have been validated for sensitivity, accuracy and cross-reactivity with 27 different cytokines, linearity of dilution and inter-/intra-assay precision. Minimal cross-reactivity was observed. Accuracy (spike recovery was between 33(IL-13) and 162% (APRIL) in human serum.
The neutralizing antibody assay was also validated for sensitivity, cross-reactivity against non-neutralizing antibody and accuracy (spike recovery 90% in serum) as well as linearity of dilution, inter- and intra-assay precision.

Human research participants

Policy information about studies involving human research participants

Population characteristics

Adult patients with advanced solid tumors starting a new cancer immunotherapy either as mono- or combination therapy except adoptive cell therapies were included in this study. Patients were included regardless of sex. Detailed characteristics can be found in Table 1 of the manuscript.

Recruitment

Patients were identified by screening for patients with solid tumors that were to receive combinatorial immune checkpoint therapies (ICT). These patients were informed of the possibility of inclusion in out study before induction of ICT and their informed consent was obtained. Self-selection bias could have presented patients with severe disease from participating. As per German law study inclusion/exclusion criteria prohibited inclusion of non-German speakers because they would be considered unable to understand all details of the informed consent resulting in a possible bias towards excluding immigrants. Patient recruitment is outlined in the CONSORT flow chart in Figure 1A

Ethics oversight

The trial received institutional ethics review board approval at Ethics Commission I Medical Faculty Heidelberg, Heidelberg University (S-373/2020, S-207/2005,) and Ethics Commission II Medical Faculty Mannheim, Heidelberg University (2021-567).

Note that full information on the approval of the study protocol must also be provided in the manuscript.

Clinical data

Policy information about clinical studies
All manuscripts should comply with the ICMJE guidelines for publication of clinical research and a completed CONSORT checklist must be included with all submissions.

Clinical trial registration

German Clinical Trial Registry (DRKS): DRKS00022890

Study protocol

Study protocol available under the registry number at www.drks.de.

Data collection

Recruitment for this exploration cohort of the prospective study was performed between 12/2019 and 07/2021. Data and patient sample collection began at the date of first patient recruitment (12/2019) until 1.10.2021 from electronic patient health records continuously updated by the treating physicians and trial staff at the National Center for Tumor Diseases, Heidelberg, Germany.

Outcomes

The primary endpoint is radiological response to therapy for which a biomarker-based prediction model will be developed based on the immune cell composition and serum proteome and metabolome of these patients.

Secondary endpoints include the immune cell and serum proteome and metabolome composition under IT and at disease progression, grade ≥3 adverse events including infections, as well as progression-free and overall survival. Select leukocyte subsets will be tested for cytotoxicity, migration, proliferation, metabolic function and cytokine production and differentiation properties in vitro and in xenograft mouse models. The effects of patient serum factors on these properties will also be investigated

Flow Cytometry

Plots

Confirm that:

- [x] The axis labels state the marker and fluorochrome used (e.g. CD4-FITC).
- [x] The axis scales are clearly visible. Include numbers along axes only for bottom left plot of group (a 'group' is an analysis of identical markers).
- [x] All plots are contour plots with outliers or pseudocolor plots.
- [x] A numerical value for number of cells or percentage (with statistics) is provided.

Methodology

Sample preparation	Serum samples were thawed and immediately analyzed in duplicates using the Legendplex Cytokine Storm Panel 1 (741091, Biolegend, CA, USA), Cytokine Storm Panel 2 (741142, Biolegend, CA, USA) or SARS-CoV-2 Neutralizing Antibody Assay (741127, Biolegend, CA, USA) according to manufacturer's instructions (25ul detection antibody per sample) and analyzed on a BD FACS Canto II flow cytometer (BD, NJ, USA). Analyte concentrations were interpolated from a standard curve using 5 parameter logistic regression using Legendplex Software v 2021.07.01. Antibodies were validated by the manufacturer but not by our laboratory. The manufacturer does not disclose clone numbers for this assay.
Instrument	BD FACS Canto II
Software	BD FACS DIVA v8.0, Legendplex Software v 2021.07.01.
Cell population abundance	no cellular data was analyzed in this study
Gating strategy	Gating on detection beads was performed according to manufacturer instructions Legendplex Cytokine Storm Panel 1 (741091, Biolegend, CA, USA), Cytokine Storm Panel 2 (741142, Biolegend, CA, USA) or SARS-CoV-2 Neutralizing Antibody Assay (741127, Biolegend, CA, USA) .

- [x] Tick this box to confirm that a figure exemplifying the gating strategy is provided in the Supplementary Information.
